# Classification and Prognostic Stratification Based on Genomic Features in Myelodysplastic and Myeloproliferative Neoplasm- and Their Overlapping Conditions

**DOI:** 10.3390/cancers16234121

**Published:** 2024-12-09

**Authors:** Jong-Mi Lee, Ginkyeng Lee, Taeksang Kim, Ari Ahn, Jin Jung, Yoo-Jin Kim, Silvia Park, Daehun Kwag, Sung-Eun Lee, Sung-Soo Park, Tong-Yoon Kim, Bin Cho, Nack-Gyun Chung, Jae Wook Lee, Jae Won Yoo, Suejung Jo, Yonggoo Kim, Myungshin Kim

**Affiliations:** 1 Department of Laboratory Medicine, College of Medicine, The Catholic University of Korea, Seoul 06591, Republic of Korea; jongmi1226@catholic.ac.kr (J.-M.L.); jiinj@catholic.ac.kr (J.J.); yonggoo@catholic.ac.kr (Y.K.); 2 Catholic Genetic Laboratory Centre, College of Medicine, The Catholic University of Korea, Seoul 06591, Republic of Korea; 3 PuzzleAI Corp., Seoul 06536, Republic of Korea; gin_lee@puzzle-ai.com (G.L.); voidbag@gmail.com (T.K.); 4 Department of Laboratory Medicine, Incheon St. Mary’s Hospital, College of Medicine, The Catholic University of Korea, Seoul 06591, Republic of Korea; 0124ari@catholic.ac.kr; 5 Department of Hematology, Catholic Hematology Hospital, Seoul St. Mary’s Hospital, College of Medicine, The Catholic University of Korea, Seoul 06591, Republic of Korea; yoojink@catholic.ac.kr (Y.-J.K.); silvia.park@catholic.ac.kr (S.P.); kdh@catholic.ac.kr (D.K.); lee86@catholic.ac.kr (S.-E.L.); sspark@catholic.ac.kr (S.-S.P.); 6 Department of Hematology, Catholic Hematology Hospital, Yeouido St. Mary’s Hospital, College of Medicine, The Catholic University of Korea, Seoul 06591, Republic of Korea; tyk@catholic.ac.k; 7 Division Hematology/Oncology, Department of Pediatrics, Seoul St. Mary’s Hospital, College of Medicine, The Catholic University of Korea, Seoul 06591, Republic of Korea; chobinkr@catholic.ac.kr (B.C.); cngped@catholic.ac.kr (N.-G.C.); dashwood@catholic.ac.kr (J.W.L.); hoiring0209@gmail.com (J.W.Y.); j.crystal1107@gmail.com (S.J.)

**Keywords:** genomic data, myeloproliferative neoplasm, myelodysplastic neoplasm, aplastic anemia

## Abstract

**Background/Objectives**: Myeloid neoplasms encompass a diverse group of disorders. In this study, we aimed to analyze the clinical and genomic data of patients with myeloproliferative neoplasm (MPN), myelodysplastic neoplasm (MDS), and their overlapping conditions, such as MDS/MPN and aplastic anemia (AA), to help redefine the disease classification. **Methods**: Clinico-genomic data of 1585 patients diagnosed with MPN (*n* = 715), MDS (*n* = 698), MDS/MPN (*n* = 78), and AA (*n* = 94) were collected. Patterns of 53 recurrent genomic abnormalities were compartmentalized into 10 groups using a Dirichlet process (DP). **Results:** These genomic groups were correlated with specific genomic features, survival outcomes, and disease subtypes. Groups DP1 and DP5, characterized by *JAK2* and *CALR* mutations, respectively, showed very favorable prognoses among the patients with MPN. Groups DP2, DP7, and DP9 demonstrated very adverse prognoses across the disease subtypes. DP2 included patients with MDS harboring *TP53* mutations and complex karyotypes; DP9 comprised patients with acute myeloid leukemia-related mutations, including *NPM1*; and DP7 included patients with *SETBP1* mutations. Groups DP10 and DP8, linked to *SF3B1* and *DDX41* mutations or chromosome 1q derivatives, presented a favorable risk profile. Improved survival was observed with transplantation in groups DP2, DP7, and DP9. **Conclusions**: These findings highlight the role of genomic classifications in guiding personalized treatment strategies, ultimately enhancing the understanding and management of myeloid neoplasms.

## Simple Summary

We aimed to enhance the classification and prognostic prediction of myeloid neoplasms, which encompass diverse disease subtypes. Unsupervised clustering identified 10 genomic groups within myeloid neoplasms that correlated with distinct disease subtypes and outcomes. Mutations in *TP53*, *NPM1*, and *SETBP1* were linked to poor prognoses, whereas *SF3B1*, *DDX41*, and 1q derivatives were associated with favorable outcomes. These findings highlight specific genetic mutations related to either favorable or adverse outcomes, suggesting new approaches to personalize treatment strategies, such as stem cell transplantation, based on a patient’s genomic profile. This approach could enhance the understanding and management of myeloid neoplasms, potentially influencing future research and clinical practices.

## 1. Introduction

Recent genomic studies introduced an alternative paradigm for the classification of myeloid neoplasms (MNs), focusing on shared and distinct genomic patterns [1,2,3]. Landmark studies on myeloproliferative neoplasm (MPN) [4], myelodysplastic neoplasm (MDS) [5,6], and their overlapping diseases [7] demonstrated the potential of genomic classification systems for disease subclassification and personalized prognostication. This has led to a fundamental shift toward genetics-based approaches in characterizing these diseases, with recent classification systems increasingly incorporating genomic attributes to align more closely with their underlying biology [2,8]. Nevertheless, current frameworks still prioritize clinical features over biological characteristics in distinguishing the major classes of MNs [3,8]. The morphological and phenotypic criteria have subjective elements, leading to inter-observer variability in diagnoses [5]. This results in biological heterogeneity within disease groups and hinders gaining insights into pathogenic mechanisms that traverse boundaries. Additionally, the diagnostic thresholds for clinical and morphological features are not sufficiently discriminative, as they often reflect the outcomes of driver genetic variations [9]. Moreover, molecular markers are intermingled in both MDS and MPN, and a comprehensive genomic characterization is still lacking. Therefore, the current system often faces challenges when applied to patients with ill-defined phenotypes, such as admixed features of myelofibrosis, dyspoiesis, and an increased blast percentage at the advanced phase.

Herein, we present the first comprehensive genomic analysis that encompassed a broad range of MNs in a Korean population and involved profiling frequently mutated genes and cytogenetic abnormalities in 1585 patients. Through unsupervised genomic grouping, we identified novel subgroups that reflected fundamental disease relationships and reinforced pre-existing disease categories to enable the enhanced individualized prediction of clinical outcomes. This approach facilitates the proposal of an integrated classification system based on genetics rather than subjective clinical criteria. In this study, we aimed to contribute to the development of a more refined classification system based on underlying genomic features.

## 2. Materials and Methods

### 2.1. Study Cohort

Clinical and genomic data were collected from the Clinical Data Warehouse of Catholic Medical Center in Seoul, Republic of Korea (http://cohort.cmcnu.or.kr, accessed on 25 December 2021). De-identified data of patients who underwent NGS testing for somatic mutations associated with MNs at diagnosis were extracted (Supplementary Table S1). Patients clinically suspected of having MPN, MDS [including clonal cytopenia of undetermined significance (CCUS) and MDS with features of aplastic anemia (AA)], and MDS/MPN were included. Accordingly, the patients initially thought to have MNs, but later confirmed histopathologically to have AA were also included. A total of 1865 NGS results were collected from 2017 to 2021. After a thorough review, diagnoses were made according to the 2022 WHO criteria, and patients with non-targeted disease or insufficient clinical data for a complete blood count, bone marrow findings, or karyotypes were excluded. Finally, the data of 1585 patients were included in modeling (Supplementary Table S2). In addition, a validation cohort of 150 patients was sequentially collected during 2022 (Supplementary Table S3). The institutional review board of the Seoul St. Mary’s Hospital, College of Medicine, The Catholic University of Korea, Seoul, Republic of Korea, approved the studies (KC22RISI0217, date of approval: 5 April 2022). Patient consent was waived owing to the retrospective nature of this study.

### 2.2. Genomic Data

DNA samples from peripheral blood or bone marrow at diagnosis were used. Mutation data were obtained using targeted NGS panels as previously described [10,11]. We used two versions of the NGS panel depending on the accessed time points. As 87 genes were common between the NGS panels, we investigated the results further. The pathogenicity of the detected variants was determined using the AMP [12] and ClinGen criteria [13]. Cytogenetic data were obtained from G-banding and selective FISH tests. Detailed methods are described in the Supplementary Materials [10,11,13,14].

### 2.3. Statistics to Analyze the Genomic Data

The detailed methods are described in Section 3, “Statistics”, of the Supplementary Materials. We performed Fisher’s exact test using the scipy library (version 1.11.3) in Python to assess the statistical significance of co-occurrence and mutually exclusive relationships between the genetic abnormalities [15]. We employed Bayesian network analysis with the bnlearn package (version 0.8.0) to infer genomic associations and potential causal relationships (https://pypi.org/project/bnlearn/0.8.0/, accessed on 19 March 2024). To define genomic subgroups, we applied the Dirichlet process (DP) using a dedicated R package (https://doi.org/10.1198/016214506000000302, accessed on 18 March 2024), which allows for flexible clustering without the need to pre-specify the number of groups, thereby capturing the heterogeneity of genomic profiles [16]. After establishing the DP groups, we applied pairwise association analysis and Bayesian network modeling to achieve a detailed genomic characterization of each DP group. The Bradley–Terry model was used to investigate the timing of the mutation acquisition through pairwise comparisons of clonal fractions, utilizing the BradleyTerry2 package (version 1.0-8) in R. For multiple testing corrections, we applied the multipletests function of the statsmodels module (version 0.14.0) in Python to ensure rigorous control of the false discovery rates.

### 2.4. Additional Statistical Tests

Survival analyses were conducted using the Kaplan–Meier method, which estimates the probability of survival over time. We employed the log-rank test to assess the differences in the survival rates between various subgroups. The overall survival was defined as the time from diagnosis to the last follow-up or death from any cause. The data of patients who underwent hematopoietic stem cell transplantation (HSCT) were censored at the time of the transplant procedure. Univariate and subsequent multivariate Cox proportional hazard (PH) models were applied to compare the clinical and hematological characteristics between different genomic subgroups.

## 3. Results

### 3.1. Clinical Characteristics of the Cohort

**Figure 1:**
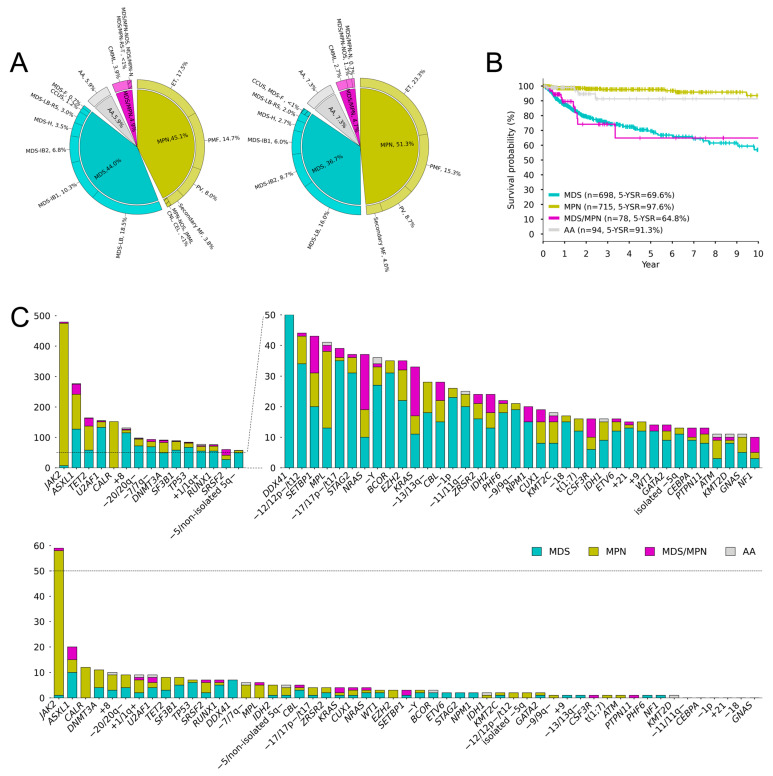
Main characteristics of the entire cohort. (**A**) Pie charts delineate the morphological subtypes of the training set (left) and validation set (right). (**B**) Survival curves of the main subtypes. (**C**) Frequency of mutations and chromosomal abnormalities of the training set (top) and validation set (bottom).

The training cohort consisted of 715 patients with MPN, 698 patients with MDS (including 19 with CCUS), 78 patients with MDS/MPN, and 94 patients with AA. The MPN comprised polycythemia vera (PV, *n* = 127), essential thrombocythemia (ET, *n* = 278), primary myelofibrosis (PMF, *n* = 233), secondary MF (*n* = 60), chronic neutrophilic leukemia (CNL, *n* = 4), juvenile myelomonocytic leukemia (JMML, *n* = 5), chronic eosinophilic leukemia (CEL, *n* = 1), and MPN not otherwise specified (NOS, *n* = 7). The patients with MDS included those with MDS with a low blast percentage (LB, *n* = 294), LB and ring sideroblasts (LB-RS, *n* = 48), hypoplastic MDS (*n* = 55), increased blast-1 percentage (IB-1, *n* = 163), IB-2 (*n* = 108), and IB-fibrosis (*n* = 11). The patients with MDS/MPN included those with chronic myelomonocytic leukemia (CMML, *n* = 62), MDS/MPN with neutrophilia (MDS/MPN-N, *n* = 7), MDS/MPN-NOS (*n* = 8), and MDS/MPN-RS and thrombocytosis (MDS/MPN-RS-T, *n* = 1). The validation cohort consisted of 77 patients with MPN, 55 patients with MDS, 7 patients with MDS/MPN, and 11 patients with AA (Figure 1A). In the survival analysis of the training cohort, patients with MPN showed the most prolonged survival, with an estimated survival time of 14.8 years (95% CI: 14.0–15.6), followed by those with AA (12.4 years, 10.0–14.8), MDS (8.6 years, 7.5–9.6), and MDS/MPN (5.7 years, 4.4–6.9) (Figure 1B). Detailed demographics and hematological and clinical features are summarized in Supplementary Tables S3 and S4 and Supplementary Figures S1 and S2.

### 3.2. Profiles of Gene Mutations and Chromosomal Abnormalities

Among the patients in the training cohort, 1271 patients (80.2%) presented with one or more mutations, with a median mutation number of 2 (range: 1–8). The average mutation number per patient was the highest for MDS/MPN (3.2 ± 1.9), followed by MPN (1.9 ± 1.7), MDS (1.7 ± 1.6), and AA (0.2 ± 0.5). Mutations were identified in 82 of 87 genes, and 36 genes were mutated in more than 1% of patients. Among them, *JAK2* (42.2%), *ASXL1* (18.4%), *TET2* (13.1%), *U2AF1* (10.0%), *CALR* (9.7%), *TP53* (8.7%), *DNMT3A* (6.0%), *SF3B1* (5.9%), and *RUNX1* (5.0%) were frequently mutated. The germline analysis confirmed germline mutations in *DDX41* in 62 patients (3.9%). A normal karyotype was observed in 1066 patients (67.3%), and 17 types of chromosomal events were identified in more than 1% of them, including +8 (8.3%), −20/20q− (6.2%), −7/7q− (5.8%), +1/1q+ (4.8%), and −5q/non-isolated 5q− (3.6%). By disease types, any chromosomal abnormality was observed in 376 patients with MDS (53.9%), 20 patients with MDS/MPN (25.6%), 108 patients with MPN (15.1%), and 15 patients with AA (16.0%). The average number of chromosomal abnormalities per patient was the highest for MDS (2.1 ± 4.0), followed by MDS/MPN (0.6 ± 1.3), MPN (0.4 ± 1.3), and AA (0.2 ± 0.5).

Next, we focused on the 53 genomic abnormalities that occurred in more than 1% of patients. Consequently, the genomic data of 1359 patients were used for further genomic grouping (Figure 1C and Supplementary Figure S2).

### 3.3. Discovering Exclusive and Co-Occurrence Patterns of Gene Mutations and Chromosomal Abnormalities

Pairwise association analysis (Supplementary Figure S3) and Bayesian network analysis (Supplementary Figure S4) revealed patterns of co-occurrences and exclusive pairings among gene mutations and chromosomal abnormalities. *JAK2* mutations were strongly and exclusively associated with *CALR* and *DDX41* mutations, as well as chromosomal abnormalities, such as +8, −5/non-isolated 5q−, and −17/17p−/t17. *CALR* mutations were exclusively associated with *U2AF1*. *TP53* mutations showed significant associations predominantly with chromosomal abnormalities: −5/non-isolated 5q−, −7/7q−, and −18. *NPM1* mutations commonly co-occurred with *BCOR* and *RUNX1*. *SETBP1* mutations frequently co-occurred with *CSF3R* and were often found with *GATA2*, *NRAS*, *NF1*, and *PTPN11* mutations. *SRSF2* mutations showed moderate associations with *CUX1*, *IDH2*, and *STAG2*.

### 3.4. Defining Genomic Groups and Their Genomic Profiles and Clonal Structures

**Figure 2:**
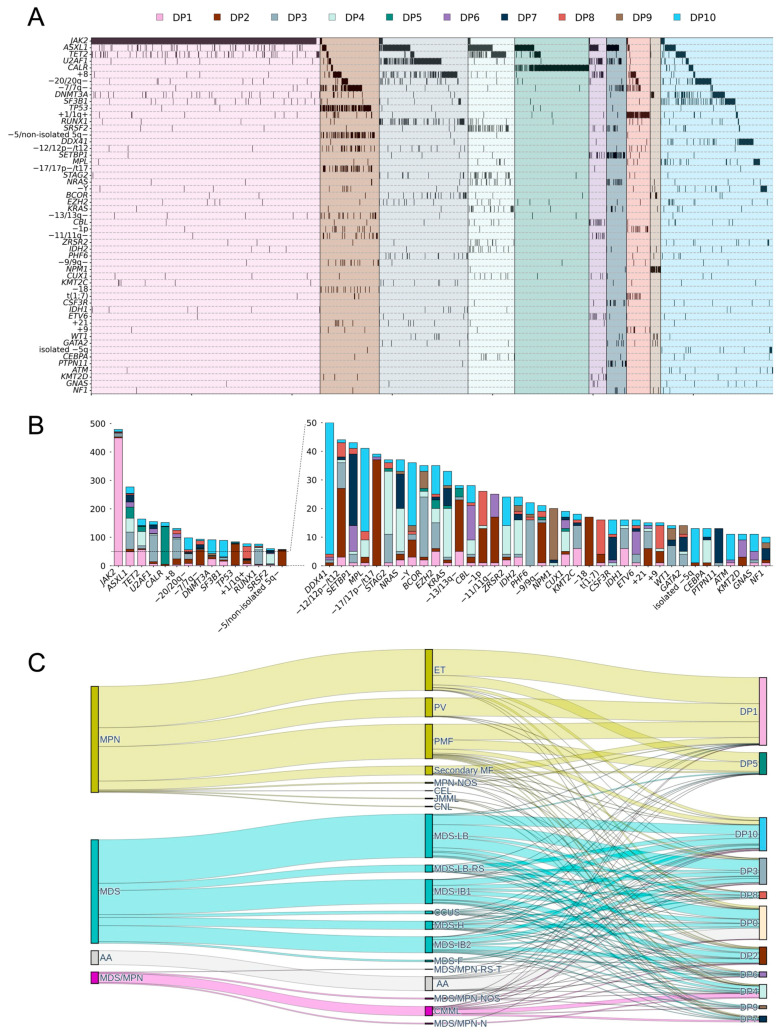
Genomic grouping of the myeloid neoplasm (MN) cohort. (**A**) Genomic groups defined by the Dirichlet process (DP) and their molecular features. (**B**) Frequency of mutations and chromosomal abnormalities broken down by genomic groups. (**C**) Sankey diagram showing relationships between morphological subtypes and genomic groups.

Using the DP, we identified 10 distinct genomic groups, each characterized by unique features (Figure 2 and Supplementary Figures S5 and S6). DP1 was the largest genomic group, which contained 456 patients characterized by activating mutations in *JAK2*. DP5 comprised 148 patients with *CALR* mutations. DP2 (*n* = 118) was characterized by *TP53* mutations and/or complex karyotypes that frequently involved −5/5q− (not isolated −5q), −7/7q−, −17/17p−/t17, −9/9q−, and −13/13q−. DP9 consisted of 21 patients with AML-like mutation patterns, specifically *NPM1* and co-occurring *BCOR*, *IDH2*, *DNMT3A*, and *WT1* mutations. DP7 (*n* = 40) showed a higher frequency of *SETBP1* mutations with co-occurring *CSF3R* and/or RAS pathway-related gene mutations. DP4 (*n* = 93) displayed a higher frequency of mutations in *TET2* and *SRSF2*, with co-occurring *STAG2*, *NRAS*, *KRAS*, *ZRSR2*, and *IDH2*. DP3 (*n* = 177) was associated with *U2AF1*, *RUNX1*, *BCOR*, and *PHF6* mutations, and chromosomal changes, such as +8. DP6 (*n* = 35) was linked to mutations in *CBL*, *ETV6*, and *KMT2D*. DP10 was the second-largest group (*n* = 224), which showed marked genomic heterogeneity, associated with mutations in *SF3B1* or *DDX41*. *DDX41* (*n* = 46) was exclusive to *SF3B1* (*n* = 58), and most *DDX41* cases (*n* = 43) accompanied germline *DDX41* mutations. Rare cases of isolated −5q, −Y, or *MPL* mutations were also included. DP8 (*n* = 47) was notably associated with chromosomal abnormalities, including isolated +1/1q+ and various derivative chromosomes that involved chromosome 1. The most prevalent common derivative chromosome identified was derivative (1;7), which accounted for 33% of cases. Additionally, derivative chromosome 1 was observed to involve other chromosomes, including chromosomes 6, 12, 13, 19, 10, 14, 15, and 22. Lastly, we assigned patients without specific genomic changes to DP0 (*n* = 226).

Bradley–Terry modeling was used to estimate the mutation acquisition order of each genomic group (Supplementary Figure S7). Mutations that drove MPN, specifically *JAK2*, *CALR*, and *MPL*, were acquired early in genomic groups DP1, DP5, and DP10. Conversely, the *JAK2* mutation occurred relatively later in the other groups, DP3, DP4, and DP6. In DP2, *TP53* mutations occurred earlier than the epigenetic regulator (*TET2*, *ASXL1*, and *DNMT3A*) and splicing factor mutations (*U2AF1* and *SF3B1*). Conversely, the epigenetic regulator (*BCOR* and *EZH2*) and splicing factor mutations (*ZRSR2* and *U2AF1*) were acquired early in the disease development process in the DP3 and DP6 groups. In DP4, mutations in the signaling pathways, such as *CBL*, *KRAS*, *NRAS*, *JAK2*, and *CSF3R*, evolved at the late stage.

### 3.5. Clinical Characteristics of the Defined Genomic Groups

In DP1, which was enriched with *JAK2* mutation, most patients with PV characterized by erythrocytosis and/or panmyelosis were included. Conversely, DP5, enriched with *CALR* mutations, predominantly included patients with ET presenting with isolated thrombocytosis. In DP2, enriched with *TP53* mutations and complex karyotypes, most patients were diagnosed with MDS that exhibited significant thrombocytopenia and increased blast percentages. However, the DP9 group predominantly included patients diagnosed with MDS, who were reclassified as having AML according to the updated WHO classification criteria [3] owing to the presence of *NPM1* mutations. In DP7, enriched with *SETBP1* and *ASXL1* mutations, the patients typically presented with hypercellular BM and could exhibit neutrophilia and/or monocytosis. Diagnoses in this group included MDS; CMML; PMF; and other MPN, such as CNL and JMML. DP4, enriched with *TET2* and *SRSF2* mutations prevalent in MDS and CMML, was characterized by relatively older age and monocytosis. DP3 and DP6 predominantly included patients diagnosed with MDS, irrespective of the blast percentage. The patients in DP6 typically presented with hypercellular BM, and some cases of PMF were also observed. In DP10, the patients enriched with *SF3B1* mutations were of older age and exhibited ring sideroblasts. Many patients were diagnosed with MDS-LB-RS, and all patients with MDS/MPN-RS-T were assigned to DP10. Additionally, the patients with other MDS and PMF were included in DP10, particularly those enriched with *DDX41* mutations. Most patients in group DP8, enriched with chromosome 1q abnormalities, were diagnosed with MDS and exhibited an increased mean corpuscular volume. Lastly, group DP0, which showed no genetic abnormalities, mostly consisted of patients diagnosed with AA and hypoplastic MDS. These patients were characterized by a younger age and hypocellular BM (Supplementary Figure S8).

### 3.6. Prognostic Implications of Genomic Risk Categories in Myeloid Neoplasms: Predicting Survival and Transplant Responses

**Figure 3:**
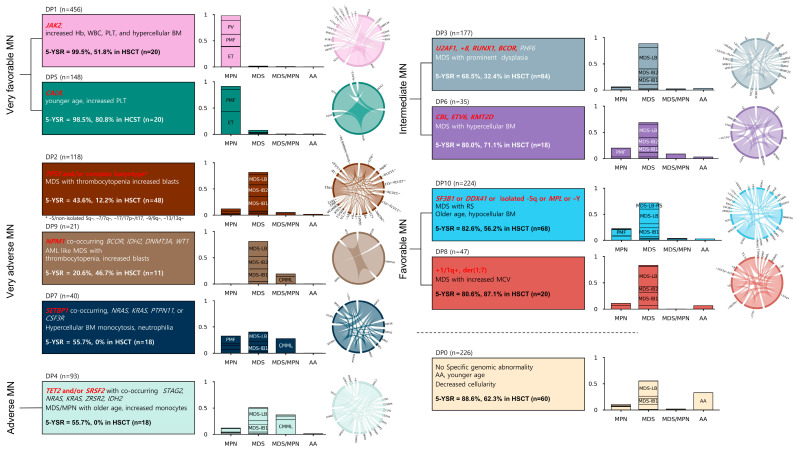
Risk stratification based on the genomic groupings in MNs. Very favorable MNs included the genomic groups DP1 and DP5, which predominantly featured *JAK2* or *CALR* mutations associated with MPN. Very adverse MNs were marked by *TP53*, *NPM1*, or *SETBP1* mutations in the genomic groups DP2, DP9, and DP7. The adverse risk group contained DP4 with *TET2* and/or *SRSF2* mutations, while intermediate MNs consisted of DP3 and DP6, characterized by MDS with *U2AF1*, +8, *RUNX1*, *BCOR*, *CBL*, *ETV6*, and *KMT2D* mutations. Favorable MNs included DP10 and DP8, marked with *SF3B1* or *DDX41* mutations or derivative chromosome 1. The extra group, named DP0, consisted of patients with no detectable genomic changes. Circos plots depict the genomic relationships within each group. Stacked bar plots display the frequencies of morphological diagnoses. Kaplan–Meier plots on the left and right show the survival probabilities for each genomic group and the patients after HSCT, respectively. MN—myeloid neoplasm, DP—Dirichlet process, MPN—myeloproliferative neoplasm, MDS—myelodysplastic neoplasm.

The 5-year survival rate (5-ysr) varied markedly across the genomic groups. DP1 and DP5 showed the highest 5-ysrs, at 99.5% and 98.5%, respectively. Conversely, DP2 and DP9 had the lowest 5-ysrs, at 43.6% and 20.6%, respectively. DP7 had a 55.7% 5-ysr, closely followed by DP4, at 57.6%. Moderate 5-ysrs were observed in DP3 and DP6, at 68.5% and 80.0%, respectively, whereas DP10 and DP8 had higher 5-ysrs of 82.6% and 80.6%, respectively. In DP10, *DDX41* and *SF3B1* showed indifferent survival outcomes (*p* = 0.3832). Finally, DP0 showed a 5-ysr of 88.6%. Thereafter, we stratified the 10 DP groups into six distinct risk categories, based on survival probabilities (Figure 3) and hazard ratios (Supplementary Figure S9). The very favorable MN group, which comprised DP1 and DP5, showed an estimated survival of 15.8 years (95% CI: 15.8–16.3). In contrast, DP2, DP7, and DP9, categorized as very adverse MNs, demonstrated a notably lower median survival of 2.2 years (95% CI: 1.6–7.4). DP4, classified as adverse MNs, had a median survival of 5.1 years (95% CI: 2.8–6.9). The intermediate MN group, which encompassed DP3 and DP6, had an estimated survival of 10.4 years (95% CI: 8.5–12.2). The favorable MN group, which consisted of DP10 and DP8, exhibited an estimated survival of 11.4 years (95% CI: 10.0–12.7). Additionally, DP0, which lacked specific genomic abnormalities, exhibited an estimated survival of 14.1 years (95% CI: 13.2–15.0).

We further assessed the risk categories, along with the clinico-hematological features using the Cox proportional hazard (PH) model. The results revealed that the very adverse MNs had the most significant impact on prognosis, where it exhibited the highest hazard ratio of 2.93 (95% CI: 1.35–6.37). Additionally, age, levels of hemoglobin and platelets, percentage of monocytes and BM blasts, cellularity, and the number of chromosomal abnormalities were observed to be significantly associated with the overall survival, although to a lesser degree than the risk categories (Table 1).

We also found that approximately 50% of the very adverse MN cases underwent HSCT (41.5% in DP2, 52.4% in DP9, and 47.5% in DP7), whereas the very favorable MN group had a limited number of patients who underwent transplantation (5.0% in DP1 and 13.5% in DP5). Our defined risk categories continued to show distinct prognostic impact, specifically in patients who did not undergo transplantation. Meanwhile, the risk categories were unable to stratify the post-transplant outcomes in patients who underwent HSCT (Supplementary Figures S9 and S10). When we compared the survival rates of patients who underwent transplantation and those who did not undergo transplantation without censoring at the time of HSCT, the survival improvement was pronounced in the very adverse MN group (Supplementary Figures S11 and S12).

### 3.7. Independent Validation of the Genomic Groups

We categorized patients from a prospectively generated independent validation cohort (Figure 3) and assigned 143 (95.3%) patients to each category as follows: DP1 (*n* = 58), DP0 (*n* = 17), DP10 (*n* = 14), DP3 (*n* = 12), DP2 (*n* = 11), DP5 (*n* = 11), DP4 (*n* = 5), DP6 (*n* = 5), DP8 (*n* = 5), DP7 (*n* = 3), and DP9 (*n* = 2). The very adverse MN group showed a significantly higher death rate than the very favorable MN (HR 15.63) and favorable MN groups (HR 14.64) (Supplementary Figure S10). The Cox PH model was unavailable for the validation cohort owing to the limited number of patients.

## 4. Discussion

This study represents an exploration of the genomic landscape of MNs within a Korean cohort by analyzing an extensive dataset of 1585 patients. Moving beyond the scope of previous research, which targeted specific MN categories [2,4,5,7,9], our study encompassed a broad spectrum of the disease, where we unveiled a comprehensive understanding of its genomic diversity. This approach not only broadened the existing knowledge base but also set a new benchmark for future genomic analyses of MNs.

To address the genetic heterogeneity of MNs, we employed the hierarchical DP, which utilizes mutations and cytogenetic changes as key components [16] and has emerged as a popular tool for clustering genomic subgroups across various MNs [17,18], facilitating a shift toward genomic classification. Through this approach, we identified 11 genomic subgroups, ranging from DP1 to DP10, where each one exhibited unique genomic characteristics, alongside a distinct DP0 that lacked these characteristics. Certain subgroups, notably DP1 and DP5, were distinguished by their unique mutational profiles—*JAK2* in DP1 and *CALR* in DP5—and associated clinical and laboratory features of MPN, which led to a very favorable prognosis. Approximately 18.7% of patients with MPN (134/715) were not classified into DP1 or DP5 but were instead grouped within MDS-related DP groups. Notably, these patients presented with MF phenotypes, suggesting a close association between MF and secondary genetic changes [4].

Conversely, other subgroups, such as DP2, were not confined to a specific disease entity and encompassed a range of conditions, including MDS irrespective of the blast percentage, MPN, MDS/MPN, and even AA. These conditions are characterized by the presence of *TP53* mutations and/or complex karyotypes and are associated with a very adverse prognosis. We further clarified the biological and prognostic importance of *NPM1* mutations, now classified as a distinct AML entity, that were applicable regardless of the blast percentage in both MDS and AML [3,8,19]. Although limited to 21 cases, individuals classified within DP9 stood out from the other subgroups owing to their very adverse outcomes, indicating the need for a distinct therapeutic strategy for MDS with *NPM1* [20,21]. Additionally, the discovery of subgroup DP9, which spanned a variety of MNs beyond MDS to include MDS/MPN, validated the recent fifth WHO classification and underscored the need for its expanded use in disease classification. Subgroup DP7, distinguished by *SETBP1* mutations, has been associated with leukemic evolution in progenitors harboring *ASXL1*, *NRAS*, and *CSF3R* mutations via specific pathways [22,23,24], indicating a very adverse prognosis. This subgroup appeared across various MNs, including MDS, CMML, CNL, and JMML. Beyond delineating these three subgroups (DP2, DP7, and DP9) based on genomic profiles and their association with a very adverse prognosis, our analysis suggests that patients within these groups might benefit from HSCT. DP4, characterized by *TET2* and/or *SRSF2* mutations, included a significant number of patients (*n* = 93) with MDS, MPN, and MDS/MPN that exhibited an adverse prognosis [25,26]. While the prognosis for DP4 was better than that for subgroups DP2, DP9, and DP7, it was poorer than that for the other subgroups. We also identified two subgroups: (1) DP3, characterized by mutations in *U2AF1*, +8, *RUNX1*, and *BCOR*, and (2) DP6, marked by mutations in *CBL*, *ETV6*, and *KMT2D*. The majority were classified within MDS, with a smaller proportion in MPN and even fewer in MDS/MPN, all of which exhibited an intermediate prognosis.

DP10 was characterized by various mutations associated with favorable prognoses, including *SF3B1*, *DDX41*, isolated 5q−, *MPL*, and −Y mutations. Our findings provide an additional insight that patients with *DDX41* mutations share a similar prognostic status with those harboring *SF3B1* mutations or isolated 5q−. This finding suggests a potential need to re-evaluate the current risk stratification systems to incorporate the *DDX41* status [27,28]. In addition, +1/1q+ and der(1;7) represent common chromosomal abnormalities in MNs [29,30], with der(1;7) being the most prevalent, accounting for 1.5–6% of patients with MDS and AML. While der(1;7) leads to both 7q− and 1q+, a prior study suggested categorizing der(1;7) as a distinct entity owing to its comparatively better outcomes than those observed in cases with only −7/7q− [29]. Our study built on these findings, offering additional evidence to justify classifying der(1;7) as a subgroup with a favorable prognosis. The last group, DP0, identified by the absence of specific genomic features, predominantly included cases of AA and hypoplastic MDS [31,32]. Distinguishing between hypoplastic MDS and AA presents a challenge owing to the scarcely observable cells in the BM. However, our results underscore the distinct biological underpinnings of hypoplastic MDS, affirming its similarity to AA. This insight helps bridge the conceptual gap between malignancies and immune-mediated disorders [6,33,34].

This study had certain limitations. AML cases were excluded during the study design because of the prevalence of gene fusions and well-defined mutations in many cases. However, including myelodysplasia-related AML may have offered valuable insights into the connection between genomic risk groups and increased blast counts. Additionally, the inclusion of rare and heterogeneous phenotypes, such as MDS/MPN, CCUS, and AA, may have introduced variability into this study, potentially affecting the applicability of our findings to broader clinical conditions. In terms of risk stratification, we did not aim to develop new prognostic models but rather aimed to compare outcomes across genomic subgroups. By classifying MNs based solely on genomic features, without incorporating clinical factors, our system showed a lower concordance with survival outcomes than established systems, such as IPSS-R and IPSS-M (Supplementary Figure S13). This underscores the critical role that clinical features play in outcome prediction. Furthermore, the validation cohort, derived from the same institution, was relatively small. Consequently, there is a need for multicenter, large-scale studies to further validate and expand upon our findings.

## 5. Conclusions

In conclusion, we aimed to elucidate the intricate genomic landscape underlying cytogenetic and molecular genetic abnormalities across a spectrum of overlapping MNs. We successfully identified subgroups that transcended specific disease diagnoses, instead grouping them by overarching disease categories. These subgroups not only highlight genetic events pivotal in disease development and prognosis but also inform the selection of patients who could benefit from HSCT. Our study addressed the prognostic needs of relatively uncommon disease categories, which are frequently overlooked in existing prognostication systems. Our results provide valuable insights into these lesser-studied MN categories, laying the foundation for the development of personalized therapeutic strategies.

## Figures and Tables

**Table 1:** Univariate and multivariate analysis results of clinical and genetic factors for overall survival.

Variable		Univariate			Multivariate		
		*p*	HR	95% CI	*p*	HR	95% CI
Genomic risk groups *	Very favorable MNs (DP1 and DP5 with respect to DP0)	<0.01	0.17	0.07–0.42	0.22	0.48	0.15–1.56
	Very adverse MNs (DP2, DP7, and DP9 with respect to DP0)	<0.01	8.66	4.85–15.47	<0.01	2.93	1.35–6.37
	Adverse MNs (DP4 with respect to DP0)	<0.01	4.04	2.01–8.15	0.15	1.89	0.79–4.51
	Intermediate MNs (DP3 and DP6 with respect to DP0)	<0.01	2.79	1.50–5.18	0.20	1.63	0.77–3.46
	Favorable MNs (DP8 and DP10 with respect to DP0)	0.08	1.79	0.94–3.40	0.54	0.79	0.37–1.70
Clinical subtypes *	MDS (with respect to AA)	<0.01	8.68	5.48–13.77	0.44	1.58	0.50–4.99
	MDS/MPN (with respect to AA)	<0.01	8.29	4.07–16.88	0.68	1.36	0.31–5.85
	MPN (with respect to AA)	0.04	0.43	0.15–0.98	0.52	1.59	0.39–6.43
Age at diagnosis (years)		<0.01	1.06	1.05–1.08	<0.01	1.04	1.02–1.05
Sex * (with respect to females)		<0.01	2.14	1.05–2.99	0.09	1.38	0.95–2.02
Hemoglobin (g/L)		<0.01	0.71	0.66–0.76	<0.01	0.82	0.75–0.90
Platelets (×10^9^/L)		<0.01	0.99	0.99–0.99	0.02	1.00	0.99–0.99
WBC count (×10^9^/L)		0.02	0.96	0.93–0.99	0.12	1.01	0.99–1.03
Neutrophil (%)		<0.01	0.97	0.96–0.98	0.09	0.99	0.98–1.00
Monocyte (%)		0.01	1.02	1.00–1.04	0.04	0.98	0.96–0.99
PB blast (%)		<0.01	1.09	1.06–1.13	0.62	0.98	0.92–1.05
BM blast (%)		<0.01	1.09	1.08–1.11	<0.01	1.06	1.03–1.08
BM cellularity (%)		0.24	1.01	1.00–1.01			
BM fibrosis grade		<0.01	0.70	0.57–0.86	0.85	0.97	0.72–1.32
Dyserythropoiesis *		<0.01	3.00	2.14–4.15	0.99	1.00	0.60–1.65
Dysgranulopoiesis *		<0.01	3.45	2.45–4.87	0.27	1.34	0.79–2.27
Megakaryocyte dysplasia *		<0.01	2.14	1.56–2.94	0.08	0.69	0.45–1.05
Ring sideroblasts (%)		0.27	1.00	0.99–1.02			
Chromosomal abnormality number		<0.01	1.16	1.14–1.19	<0.01	1.07	1.03–1.11
Mutation number		<0.01	1.30	1.19–1.42	0.65	1.03	0.92–1.15

* Categorical variables: HR—hazard ratio, CI—confidence interval, MN—myeloid neoplasm, MDS—myelodysplastic neoplasm, MPN—myeloproliferative neoplasm, WBC—white blood cell, PB—peripheral blood, BM—bone marrow. Variables identified to be significant prognostic factors in the univariate analysis were included in the multivariate analysis.

## Data Availability

The variant data supporting this study’s findings are included in the supplementary tables. The raw sequencing data for the validation cohort will be openly available in the NCBI SRA repository. Data not available in the repository are available upon reasonable request from the first author, J.-M.L., and the corresponding author, M.K.

## Supplementary Materials

The following supporting information can be downloaded from https://www.mdpi.com/article/10.3390/cancers16234121/s1, Supplementary Methods—Figure S1: Clinical characteristics of the study cohort (training set); Figure S2: Variant allele frequency (VAF) distribution of the driver mutations; Figure S3: Pairwise associations between the genes and cytogenetic abnormalities; Figure S4: Genomic associations based on Bayesian networks; Figure S5: Genomic profiles and posterior distributions from the hierarchical Dirichlet process (DP); Figure S6: Frequencies of the disease phenotypes in each genomic subgroup; Figure S7: Bradley–Terry model analysis of the genetic mutation sequences in each genomic cluster; Figure S8: Hematological parameters of each genomic subgroup; Figure S9: Survival probability according to the newly defined genomic subgroups (training set); Figure S10: Survival probability according to the newly defined genomic subgroups (validation set); Figure S11: Comparison of the survival probabilities between patients who underwent hematopoietic stem cell transplantation (HSCT) and those who did not undergo HSCT according to the risk categories; Figure S12: Comparison of the survival probabilities between patients who underwent hematopoietic stem cell transplantation (HSCT) and those who did not undergo HSCT according to the genomic subgroups; Figure S13: Validation of genomic risk categories compared with IPSS-R and IPSS-M in MDS; Table S1: List of genes sequenced for the entire study cohort; Table S2: Baseline characteristics of the study cohort for training; Table S3: Baseline characteristics of the study cohort for validation; Table S4: Comparison of the two study cohorts.
