# Supplementary material for: Classification and Prognostic Stratification Based on Genomic Features in Myelodysplastic and Myeloproliferative Neoplasm- and Their Overlapping Conditions
Source: Cancers (Basel). 2024 Dec 9;16(23):4121. doi: 10.3390/cancers16234121 (PMC11640283; doi:10.3390/cancers16234121)
Supplement: Supplementary file 1 [file cancers-16-04121-s001.zip › cancers-3197173-supplementary.pdf]

Supplementary Materials

Materials and methods

1. Molecular analysis

DNA was extracted from bone marrow aspirates using the QIAamp DNA Mini Kit (Qiagen, Hilden, Germany) or Qiasymphony Dsp DNA Mini Kit (Qiagen). Next generation sequencing (NGS) was performed using a customized myeloid panel (SM panel). Samples of patients diagnosed from August 2017 to October 2020 were analyzed using the first version of the SM panel, which contained 87 genes, as previously described.[1,2] For samples of patients diagnosed at a later stage, the second version of the SM panel, containing 110 genes, was used. In total, 87 genes were analyzed using both panels (Supplementary Table S1).

According to the manufacturer's instructions, target capturing sequencing was performed using a customized target kit (Agilent Technologies, 3039061 for version 1, HEMEaccuTest MDS/MPN for version 2). The enriched fragments were amplified and sequenced on HiSeq4000 (in version 1) or NextSeq550Dx (in version 2). After demultiplexing, the reads were aligned to the human reference genome hg19 (GRCh37) using BWA (0.7.10), and duplicate reads were removed with MarkDuplicates (GATK 4.0.6.0). Local realignment, score recalibration, and sequence data filtering were performed using GATK (4.0.6.0). Variants were annotated using SnpEff (4.3).

The pathogenicity of the annotated variants was determined according to the standards and guidelines of the Association for Molecular Pathology (AMP)[3] and Somatic Oncogenicity classification.[4] All variants with a minor allele frequency of >0.01 were filtered out based on the Exome Aggregation Consortium (ExAC, http://exac.broadinstitute.org/) and genome aggregation database (gnomAD, https://gnomad.broadinstitute.org/), as well as the ethnic-specific Korean Variant Archive (KOVA, http://kobic.re.kr/kova/). Variants of synonymous, intronic, or non-coding regions were further excluded. Among the remaining variants, those with more than 20 reads and 5% variant allele frequency (VAF) were considered mutants. Hot-spot mutations in driver genes with less than 5% VAF were present with a low allele burden. All mutations were manually verified using the Integrative Genomic Viewer. Germline confirmation using buccal DNA was conducted for all detected DDX41 mutations.

2. Cytogenetic study

Conventional bone marrow (BM) karyotyping was performed on G-banded metaphase chromosomes using routine techniques. Karyotypes were interpreted according to the International System for Human Cytogenetic Nomenclature 2016 and 2020. The number of cytogenetic events was considered the sum of chromosome gains, losses, partial gains, partial losses, and rearrangements. For 182 patients who presented with myelodysplastic neoplasms (MDS) features, fluorescence in situ hybridization (FISH) tests were conducted using six DNA probes: LSI ATM/CEP11 probe for detecting deletions at 11q22, LSI MYB probe for detecting deletions at 6q23, LSI D13S319/CEP13 probe for detecting deletions at 13q14, LSI TP53/CEP17 probe for detecting deletions at 17p13.1, and LSI IGH Dual Color, Break Apart Rearrangement probe and CEP12 probe for detecting trisomy 12. For these FISH tests, probes from Vysis Inc./Abbott Laboratories (Abbott Park, IL, USA) were utilized.

3. Statistics

3.1. Pairwise associations among genes and cytogenetic abnormalities

To assess pairwise association among genes and/or cytogenetic abnormalities, we calculated the co-occurrence of genomic abnormalities across patients. The number of patients showing mutation co-occurrence was quantified. Next, 2×2 contingency tables were generated by each pair and the significance was evaluated using Fisher's exact test. Furthermore, the odds ratio was calculated for each possible pairing of genes and/or cytogenetic abnormalities. Odds ratios less than 1 indicate that the mutation pairs were mutually excluded, whereas odds ratios greater than 1 imply mutation co-occurrence. Fisher_exact function from scipy Python module (version 1.11.3) to carry out the analysis [Nature Methods volume 17, pages261–272 (2020)].

3.2. Relationships of genomic abnormalities based on Bayesian Networks

We used Bayesian Networks (BNs) to define the relationships of genomic abnormalities among MDS, myeloproliferative neoplasms (MPN), and overlapping conditions in a more comprehensive manner. BNs are graph-based statistical models used to model complex probabilistic relationships. They employ directed graphs to represent conditional dependencies among variables, supporting probabilistic inference, and decision-making under uncertainty. The hill-climb search algorithm was employed to optimize the structure of the Directed Acyclic Graph (DAG) by iteratively modifying edges—adding, removing, or

reversing them—to maximize the network's score and reveal genomic dependencies. Python package bnlearn(version 0.8.0) was used for this analysis [Python package for Bayesian network analysis https://pypi.org/project/bnlearn/0.8.0/]. In the BN model, nodes represented distinct genomic abnormalities and directed edges indicated conditional dependencies.

3.3. Genomic clustering based on Hierarchical Dirichlet process

We employed a Bayesian nonparametric approach to analyze complex genomic data, specifically focusing on identifying distinct genomic subgroups within a large patient cohort. This approach was grounded in the use of a Dirichlet process mixture model (DPMM). A significant advantage of the DPMM is its inherent flexibility, allowing for the number of clusters to be inferred from the data itself, rather than being pre-specified. This characteristic makes the DPMM a versatile tool for a wide range of clustering applications.

The DPMM hinges on the Dirichlet process (DP), defined by a concentration parameter (α) and a base distribution (H). The base distribution (H) represents the underlying parameter distributions across clusters, whereas the concentration parameter (α) governs the probable number of clusters. The mathematical formulation of the DPMM is as follows: DP(α,H), with α controlling cluster abundance and H detailing the parameter space. Each cluster k is associated with a parameter θ_k, independently drawn from the base distribution H. The data points x_i are then generated from α based on the parameters θ_k of their respective clusters.

The implementation of DPMM in our study is primarily based on Markov Chain Monte Carlo (MCMC) methods, specifically Gibbs sampling, which facilitates the estimation of cluster memberships for each data point and the determination of corresponding cluster parameters. For our analysis, we utilized the R package hdp, which is publicly accessible at https://github.com/nicolaroberts/hdp. This package facilitated the Monte Carlo Markov Chain (MCMC) sampling of posterior DP. Our approach involved running the MCMC process under four distinct initial conditions, essentially creating four separate chains. In each chain, the initial 2000 iterations were discarded to mitigate the impact of initial conditions on the results. Subsequently, we collected 2000 samples, selecting every 20th iteration to reduce autocorrelation. To refine our cluster analysis, we implemented a merging criterion based on cosine similarity. Specifically, clusters exhibiting a cosine similarity above 0.8 were merged, ensuring a more robust and meaningful clustering outcome.

3.4. Bradley–Terry model

The Bradley–Terry model was used to assign the relative order of mutation acquisition for each DP component, based on pairwise relationships among patients with at least two mutations. Each genetic mutation is treated as a "competitor," and the VAF values are used to infer their relative strengths or occurrences over time. By comparing pairs of mutations (much like competitors in a tournament), the model assesses which mutation is more dominant or prevalent, which in turn suggests its relative timing in the genetic sequence. By setting a threshold where a decisive "win" in the comparative strength of mutations is determined when the difference in corrected VAF values is greater than or equal to 5, the analysis becomes more robust and specific. The R package BradleyTerry2 (version 1.0-8) was used to generate estimates of relative mutation timing and provide point estimates and 95% confidence intervals, representing the results of the Bradley–Terry model for the relative ordering of genes in their temporal acquisition.

Supplementary Table S1. List of genes sequenced for the entire study cohort

Target gene	Interval	Number of regions	Size (base pairs)
ABCA12	chr2:215797358-216002931	54	7929
ABL1	chr9:133589707-133761070	12	3529
ASXL1	chr20:30946579-31025141	17	4720
ATM	chr11:108098352-108236235	62	9171
ATRX	chrX:76763829-77041487	36	7543
ATXN7L1	chr7:105248299-105517004	15	3132
BCOR	chrX:39909169-39937182	15	5348
BRAF	chr7:140426294-140624503	21	2379
BRCC3	chrX:154299803-154348425	11	1021
CALR	chr19:13049494-13054795	10	1294
CBL	chr11:119077128-119170491	16	2721
CBLB	chr3:105377814-105588232	20	3079
CD101	chr1:117544440-117576723	9	3066
CEBPA	chr19:33792244-33793425	1	1182
CREBBP	chr16:3777719-3929917	31	7368
CSF1R	chr5:149433632-149465990	22	3003
CSF3R	chr1:36931697-36945097	16	2698
CTCF	chr16:67644736-67671775	10	2184
CUX1	chr7:101459311-101926382	34	5433
DDX41	chr5: 176938739-176943985	14	3924
DNMT1	chr19:10244343-10311559	43	5292
DNMT3A	chr2:25457148-25536853	25	2888
EGFR	chr7:55086971-55273310	31	4084
EP300	chr22:41489009-41574960	31	7245
ERG	chr21:39739557-39947624	12	1764
ETV6	chr12:11803062-12044535	10	1443
EZH2	chr7:148504738-148544390	21	2456
FBXW7	chr4:153244033-153332955	14	2618
FLT3	chr13:28578189-28674647	25	3004
GATA1	chrX:48649517-48652675	5	1346
GATA2	chr3:128199862-128205874	5	1443
GNAS	chr20:57415162-57485884	17	4096
HIPK2	chr7:139257673-139477422	16	3708
IDH1	chr2:209101803-209116275	8	1248
IDH2	chr15:90627498-90645622	11	1359

INVS	chr9:102866804-103062956	17	3395
IRF1	chr5:131819643-131825170	9	978
JAK2	chr9:5021988-5126791	23	3399
KDM2B	chr12:121867919-122018816	29	4276
KDM6A	chrX:44732798-44970656	31	4470
KIT	chr4:55524182-55604723	21	2931
KRAS	chr12:25362729-25398318	6	708
LAMB4	chr7:107664484-107763609	35	5507
MECOM	chr3:168802697-169381160	20	3816
MET	chr7:116335811-116436178	21	4359
KMT2A (MLL1)	chr11: 118307112-118392899	40	15360
KMT2C (MLL3)	chr7:151833917-152132871	62	15030
KMT2D (MLL4)	chr12: 49415518-49449172	51	21294
KMT2E (MLL5)	chr7:104681400-104753780	27	5743
MN1	chr22:28146903-28196531	2	3963
MPL	chr1:43803520-43818443	12	1993
NCOR2	chr12:124809948-124979797	49	7734
NF1	chr17:29422226-29705949	63	9011
NLRP1	chr17:5405134-5487277	18	4493
NOTCH1	chr9:139390523-139440238	34	7668
NPM1	chr5:170814953-170837569	12	894
NRAS	chr1:115251156-115258781	4	570
NUP98	chr11:3692612-3803347	35	5567
OCA2	chr15:28000534-28327020	24	2573
PDGFRA	chr4:55106220-55161439	24	3450
PHF12	chr17:27233201-27278622	17	3519
PHF6	chrX:133511648-133559360	9	1207
PRPF40B	chr12:50017374-50037975	26	2682
PRPF8	chr17:1553953-1587865	42	7151
PTPN11	chr12:112856916-112942568	16	1822
RAD21	chr8:117859739-117878968	13	1896
RAD50	chr5:131893017-131978781	27	4211
RINT1	chr7:105172763-105207758	16	2417
ROBO1	chr3:78648063-79639061	34	5223
ROBO2	chr3:75986645-77695209	32	4862
RUNX1	chr21:36164432-36421196	11	1584
RUNX1T1	chr8:92972470-93115112	20	2350
SETBP1	chr18:42281312-42643663	6	4980
SF3A1	chr22:30730583-30752781	16	2382

SF3B1	chr2:198257027-198299723	27	4045
SMC1A	chrX:53407024-53449549	26	3882
SMC3	chr10:112327575-112364060	29	3654
SRSF2	chr17:74732243-74733242	2	666
STAG2	chrX:123156478-123234447	34	3861
TET1	chr10:70332096-70451571	11	6411
TET2	chr4:106111627-106197676	10	6165
TP53	chr17:7565257-7579912	14	1378
TP53BP1	chr15:43699581-43785241	31	6130
U2AF1	chr21:44513212-44527604	9	790
U2AF2	chr19:56166471-56185434	14	1541
WT1	chr11:32410604-32456891	11	1568
ZRSR2	chrX:15808619-15841365	12	1690

Supplementary Table S2. Baseline characteristics of the study cohort for training

Variable	MDS	MPN	MDS/MPN	AA
Number of patients	698	715	78	94
Age at diagnosis (years)	58.5 (23.0–81.0)	58.0 (26.8–76.0), 0.173	66.0 (30.9–83.1), <0.01	53.0 (21.3–82.1), <0.01
Sex (Female)	39.4% (275/698)	53.1% (380/715)	39.7% (31/78)	53.2% (50/94)
Hemoglobin (g/L)	9.4 (5.8–14.4)	13.0 (7.3–19.2), <0.01	9.8 (5.8–14.6), 0.212	10.0 (6.1–15.2), <0.05
Hematocrit (%)	28.5 (17.4–42.4)	39.8 (23.0–61.0), <0.01	31.2 (18.3–44.8), <0.05	30.0 (17.9–45.5), 0.077
MCV (fl)	99.2 (81.0–117.8)	90.3 (73.5–111.8), <0.01	92.7 (78.1–113.4), <0.01	100.7 (86.2–115.9), 0.217
MCH (pg)	33.2 (25.5–39.3)	29.5 (21.9–37.9), <0.01	29.8 (23.4–36.8), <0.01	33.8 (29.1–38.4), <0.05
MCHC (%)	33.3 (29.9–35.4)	32.5 (29.6–34.6), <0.01	32.2 (29.5–34.2), <0.01	33.6 (31.1–35.8), <0.01
RBC count ($\times10^{12}$/L)	2.9 (1.7–4.6)	4.4 (2.4–7.6), <0.01	3.3 (2.0–5.2), <0.01	3.0 (1.7–4.6), 0.217
Platelets ($\times10^{9}$/L)	80.5 (9.0–366.0)	510.0 (31.8–1326.5), <0.01	112.5 (10.8–583.8), <0.01	62.5 (7.0–245.7), <0.01
WBC count ($\times10^{9}$/L)	3.1 (1.0–11.2)	9.0 (2.7–31.0), <0.01	18.0 (2.9–84.3), <0.01	3.3 (1.1–7.0), 0.25
Neutrophils ($\times10^{9}$/L)	39.0 (7.4–79.1)	66.0 (36.6–87.1), <0.01	53.2 (16.9–81.0), <0.01	42.0 (17.3–72.0), 0.122
Monocytes (%)	6.0 (0.4–28.0)	5.0 (1.0–13.0), <0.01	17.0 (1.9–49.4), <0.01	6.0 (2.0–19.7), 0.564
PB blast (%)	0.0 (0.0–6.0)	0.0 (0.0–5.1), <0.05	0.0 (0.0–8.1), <0.01	0.0 (0.0–0.7), <0.01
BM blast (%)	2.0 (0.0–17.0)	0.0 (0.0–4.0), <0.01	2.0 (0.0–13.1), 0.088	0.0 (0.0–1.7), <0.01
BM cellularity (%)	30.0 (1.0–90.0)	50.0 (1.0–100.0), <0.01	70.0 (1.0–100.0), <0.01	10.0 (5.0–24.6), <0.01
Dyserythropoiesis *	73.4% (495/674)	2.6% (12/466)	40.0% (30/75)	0% (0/85)
Dysgranulopoiesis *	72.7% (490/674)	3.2% (15/466)	92.0% (69/75)	0% (0/85)
Megakaryocyte dysplasia *	67.1% (452/674)	3.9% (18/466)	56.3% (49/75)	0% (0/85)
Myelofibrosis*	11.3% (76/674)	50.2% (234/466)	21.3% (16/75)	0% (0/85)
Ring sideroblasts (%)	0.0 (0.0–43.3)	0.0 (0.0–0.0), <0.01	0.0 (0.0–0.0), <0.01	0.0 (0.0–0.0), <0.01
Transplantation*	41.3% (288/698)	9.9% (71/715)	26.9% (21/78)	13.8% (13/94)
Abnormal karyotype*	53.9% (376/698)	15.1% (108/715)	25.6% (20/78)	16.0% (15/94)
Complex karyotype*	12.6% (88/698)	1.7% (12/715)	0% (0/78)	0% (0/94)
Number of mutations	1.0 (0.0–5.0)	2.0 (0.0–4.1), <0.01	3.0 (0.0–8.0), <0.01	0.0 (0.0–1.7), <0.01
Estimated survival year	8.6 (7.5-9.6)	14.8 (14.0-15.6)	5.7 (4.4-6.9)	12.4 (10.0-14.8)
2-year survival rate (%)	79.3 (75.2–82.8)	98.0 (96.4–99.0)	74.1 (54.9–86.1)	94.6 (83.5–98.3)
5–year survival rate (%)	69.6 (63.8–74.6)	97.6 (95.6–98.7)	64.8 (39.2–81.8)	91.3 (77.2–96.9)
10-year survival rate (%)	56.9 (47.1–65.5)	93.5 (85.5–97.1)	64.8 (39.2–81.8)	91.3 (77.2–96.9)

Continuous variables are described as median and range (2.5%–97.5%), *P* value of Mann–Whitney test using MDS as the control group.

Categorical variables are described as frequency and percentage.

MDS myelodysplastic neoplasm, *MPN* myeloproliferative neoplasm, *AA* aplastic anaemia, *MCV* mean corpuscular volume, *MCH* mean corpuscular haemoglobin, *MCHC* mean corpuscular haemoglobin concentration, *RBC* red blood cell, *WBC* white blood cell, *PB* peripheral blood, *BM* bone marrow

Supplementary Table S3. Baseline characteristics of the study cohort for validation

Variable	MDS	MPN	MDS/MPN	AA
Number of patients	55	77	7	11
Age at diagnosis (years)	62.0 (23.0–82.3)	56.0 (26.7–79.3), 0.054	56.0 (21.4–74.0), 0.688	47.0 (30.5–65.0), <0.05
Sex (Female)	63.6% (35/55)	45.5% (35/77)	42.9% (3/7)	54.5% (6/11)
Hemoglobin (g/L)	9.1 (5.0–13.7)	13.5 (7.8–17.0), <0.01	9.3 (5.7–12.2), 0.938	9.5 (4.7–14.7), 0.606
Hematocrit (%)	27.3 (15.1–42.2)	41.1 (25.0–55.8), <0.01	29.5 (17.4–37.1), 0.841	28.4 (14.5–42.0), 0.744
MCV (fl)	100.0 (81.5–113.3)	91.3 (81.8–121.9), <0.01	96.9 (89.6–107.8), 0.656	107.5 (94.1–126.8), 0.066
MCH (pg)	33.6 (24.8–38.4)	30.1 (25.4–39.2), <0.01	30.2 (29.2–35.9), 0.178	35.1 (32.2–42.8), <0.05
MCHC (%)	33.1 (30.5–34.9)	32.4 (30.1–34.4), <0.01	31.9 (30.4–33.4), <0.05	33.7 (32.1–35.2), 0.13
RBC count ($\times 10^{12}$/L)	2.7 (1.5–4.6)	4.5 (2.3–6.5), <0.01	2.8 (1.9–4.0), 0.929	2.9 (1.4–4.1), 0.731
Platelets ($\times 10^{9}$/L)	76.0 (6.4–282.8)	571.0 (78.0–1152.5), <0.01	138.0 (27.9–691.0), 0.344	63.0 (14.5–140.0), 0.182
WBC count ($\times 10^{9}$/L)	2.3 (0.8–14.5)	9.0 (3.9–21.2), <0.01	9.9 (4.5–24.6), <0.01	3.2 (2.0–4.9), 0.09
Neutrophils ($\times 10^{9}$/L)	31.0 (7.4–70.2)	66.0 (41.6–82.5), <0.01	59.0 (14.9–73.8), 0.133	49.0 (28.5–69.5), <0.05
Monocytes (%)	6.0 (1.0–25.0)	6.0 (2.0–14.3), 0.928	26.0 (9.2–47.4), <0.01	8.0 (1.5–17.2), 0.159
PB blast (%)	0.0 (0.0–9.6)	0.0 (0.0–5.4), 0.357	0.0 (0.0–2.5), 0.584	0.0 (0.0–0.0), 0.079
BM blast (%)	2.0 (0.0–16.3	0.0 (0.0–3.2), <0.01	1.0 (0.0–3.7), 0.097	0.0 (0.0–3.2), <0.01
BM cellularity (%)	30.0 (5.0–96.5)	60.0 (20.0–90.0), <0.01	70.0 (22.5–100.0), <0.01	15.0 (5.0–27.5), <0.05
Dyserythropoiesis *	80.0% (44/55)	1.8% (1/56)	71.4% (5/7)	9.1% (1/11)
Dysgranulopoiesis *	67.3% (37/55)	42.9% (3/7)	100% (7/7)	9.1% (1/11)
Megakaryocyte dysplasia *	61.8% (34/55)	42.9% (3/7)	71.4% (5/7)	9.1% (1/11)
Myelofibrosis*	21.8% (12/55)	50.0% (28/56)	42.9% (3/7)	0% (0/11)
Ring sideroblasts (%)	0.0 (0.0–46.7)	0.0 (0.0–0.0), <0.05	0.0 (0.0–0.0), 0.423	0.0 (0.0–0.0), 0.311
Transplantation*	38.2% (21/55)	9.1% (7/77)	42.9% (3/7)	9.1% (1/11)
Abnormal karyotype*	47.3% (26/55)	15.6% (12/77)	14.3% (1/7)	18.2% (2/11)
Complex karyotype*	12.7% (7/55)	0% (0/77)	14.3% (1/7)	0% (0/11)
Number of mutations	1.0 (0.0–5.3)	1.0 (0.0–5.1), 0.121	3.0 (0.2–5.8), 0.086	0.0 (0.0–1.0), <0.05
Estimated survival year	1.5 (1.3-1.6)	1.6 (1.4-1.8)	1.6 (1.5-1.8)	1.3 (1.0-1.7)
2-year survival rate (%)	90.3 (76.1–96.3)	98.6 (90.5–99.8)	68.6 (21.3–91.2)	100 (100.0–100.0)

Continuous variables are described as median and range (2.5–97.5%), *P* value of Mann–Whitney test using MDS as the control group.

Categorical variables are described as frequency and percentage.

MDS myelodysplastic neoplasm, *MPN* myeloproliferative neoplasm, *AA* aplastic anaemia, *MCV* Mean corpuscular volume, *MCH* mean corpuscular haemoglobin, *MCHC* mean corpuscular haemoglobin concentration, *RBC* red blood cell, *WBC* white blood cell, *PB* peripheral blood, *BM* bone marrow.

Supplementary Table S4. Comparison of two study cohorts

Variable	Training set	Validation set	*P*
Number of patients	1585	150	-
Age at diagnosis (years)	58.0 (24.0–79.0)	58.0 (23.4–81.3)	0.767
Sex (Female)	46.4% (736/1585)	52.7% (79/150)	0.169
Hemoglobin (g/L)	10.7 (6.1–18.0)	10.9 (5.3–16.8)	0.727
Platelets (×10⁹/L)	172.0 (12.0–1101.4)	257.5 (12.9–1030.9)	0.066
WBC Count (×10⁹/L)	5.5 (1.2–30.1)	6.5 (1.2–21.6)	0.927
2022 WHO classification (morphologically defined)			
MDS			
CCUS	19 (1.2%)	1 (0.7%)	0.733
MDS-LB	294 (18.5%)	24 (16.0%)	
MDS-LB-RS	48 (3.0%)	3 (2.0%)	
MDS-H	55 (3.5%)	4 (2.7%)	
MDS-IB1	163 (10.3%)	9 (6.0%)	
MDS-IB2	108 (6.8%)	13 (8.7%)	
MDS-F	11 (0.7%)	1 (0.7%)	
MPN			
PV	127 (8.0%)	13 (8.7%)	0.902
ET	278 (17.5%)	35 (23.3%)	
PMF	233 (14.7%)	23 (15.3%)	
Secondary MF	60 (3.8%)	6 (4.0%)	
CNL	4 (0.3%)	0 (0%)	
JMML	5 (0.3%)	0 (0%)	
CEL	1 (0.1%)	0 (0%)	
MPN-NOS	7 (0.4%)	0 (0%)	
MDS/MPN			
CMML	62 (3.9%)	4 (2.7%)	0.470
MDS/MPN-N	7 (0.4%)	1 (0.7%)	
MDS/MPN-RS-T	1 (0.1%)	0 (0%)	
MDS/MPN-NOS	8 (0.5%)	2 (1.3%)	
AA			
AA	94 (5.9%)	11 (7.3%)	1.00
2-year survival rate (%)	88.4 (86.3–90.3)	94.6 (89.0–97.4)	-
5-year survival rate (%)	83.5 (80.5–86.1)	93.4 (87.2–96.7)	-
10-year survival rate (%)	76.2 (70.6–80.8)	93.4 (87.2–96.7)	-

Continuous variables are described as median and range (2.5–97.5%), *P* value of Mann–Whitney test. Categorical variables are described as frequency and percentage and the P value of the chi-square test. *MDS* myelodysplastic neoplasm, *MPN* myeloproliferative neoplasm, *AA* aplastic anaemia

Supplementary Figure S1. Clinical characteristics of the study cohort (training set)

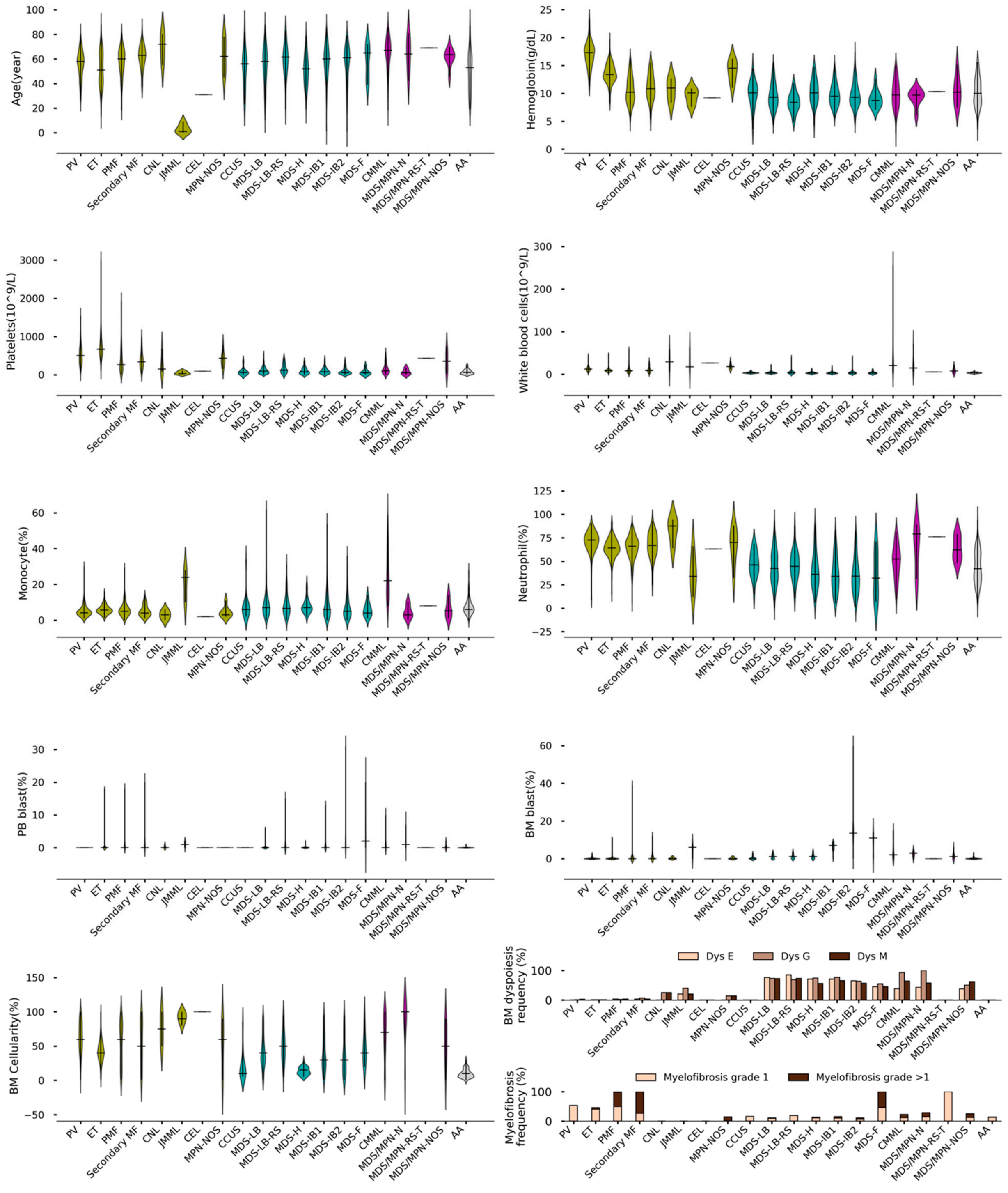

Supplementary Figure S2. Variant allele frequency (VAF) distribution of driver mutations

The boxplots reveal the spread and central values of VAFs within each category, with the median value represented by the line within each box, interquartile range denoted by the box itself, and whiskers extending to the furthest points excluding outliers, marked as individual circles.

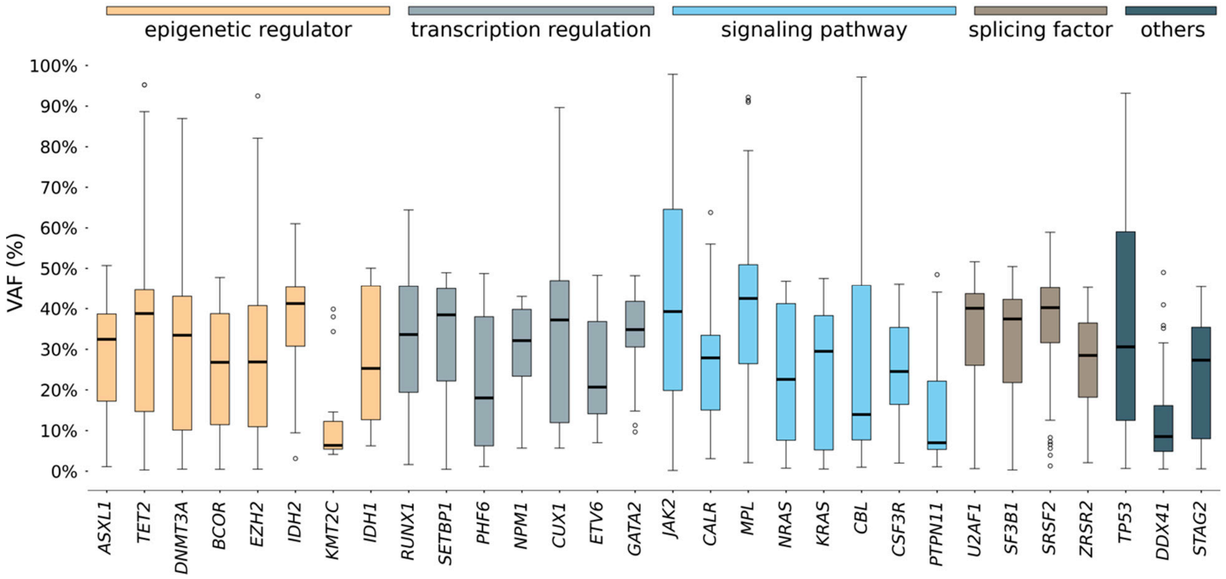

Supplementary Figure S3. Pairwise associations among genes and cytogenetic abnormalities

Pairwise associations among genes and cytogenetic abnormalities occurring at more than 1% were analyzed. In the lower triangle, the number of patients showing co-occurrences of genomic abnormalities are illustrated using gray scale. In the upper triangle, the co-occurrence and mutual exclusivity were assessed using odds ratios, and their significances were evaluated using Fisher's test with Benjamini–Hochberg correction.

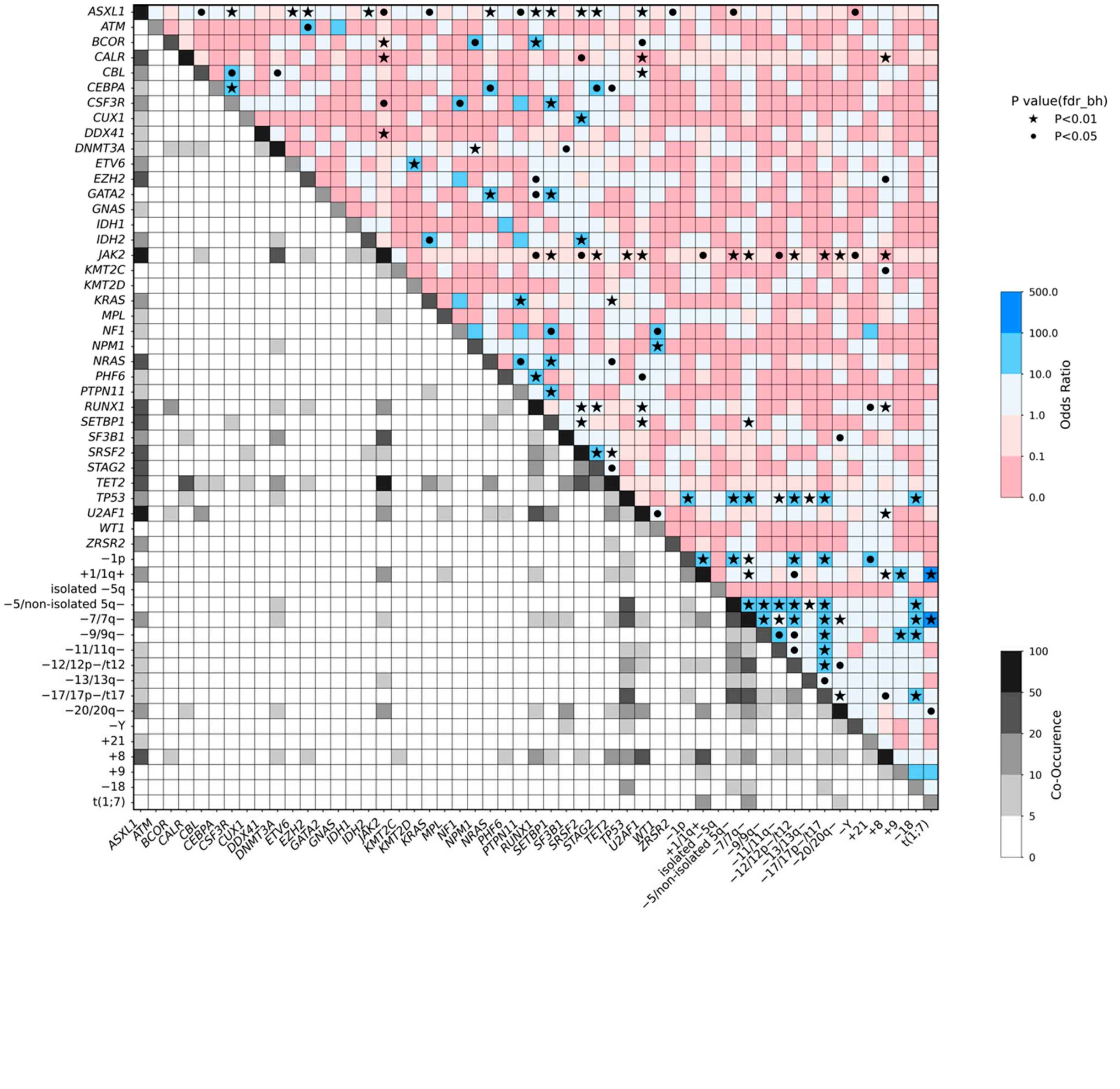

Supplementary Figure S4. Genomic associations based on Bayesian networks

The size of each node accounts for the number of corresponding genomic or cytogenetic alterations. The color of each link represents co-occurrence (odds >1, blue) or mutual exclusivity (odd <1, red). We computed edge strength using Fisher's exact test and pruned the insignificant edges (P>0.05). Edge thickness represents the value of log-odds ratios. In cases of mutually exclusive relationships, it was made proportional to the absolute value of the log-odds. Furthermore, the node colors correspond to categories mentioned in Supplementary Figure S2.

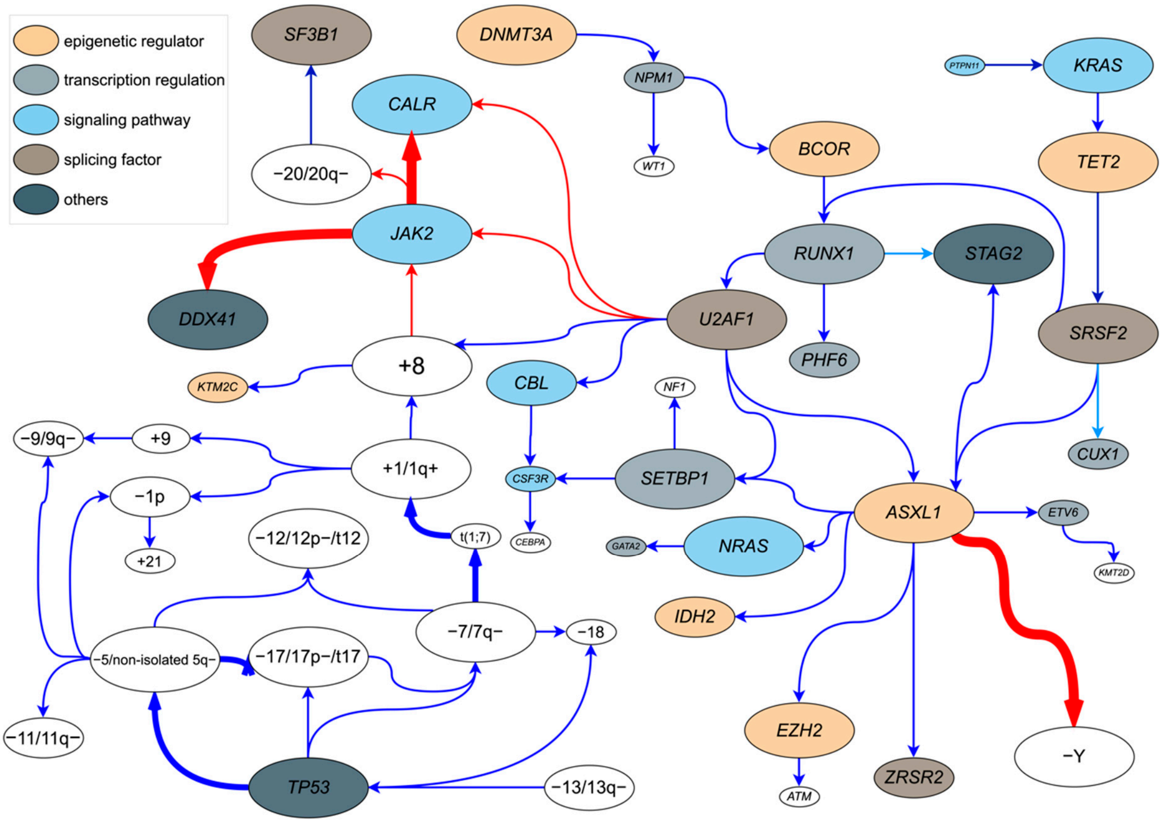

Supplementary Figure S5. Genomic profiles and posterior distributions from the hierarchical Dirichlet process (DP) Bar graphs of genomic profiles (red bars) and posterior distributions (blue bars) of 10 distinct DP groups. The x-axis of the graph spans from 0 to 1.

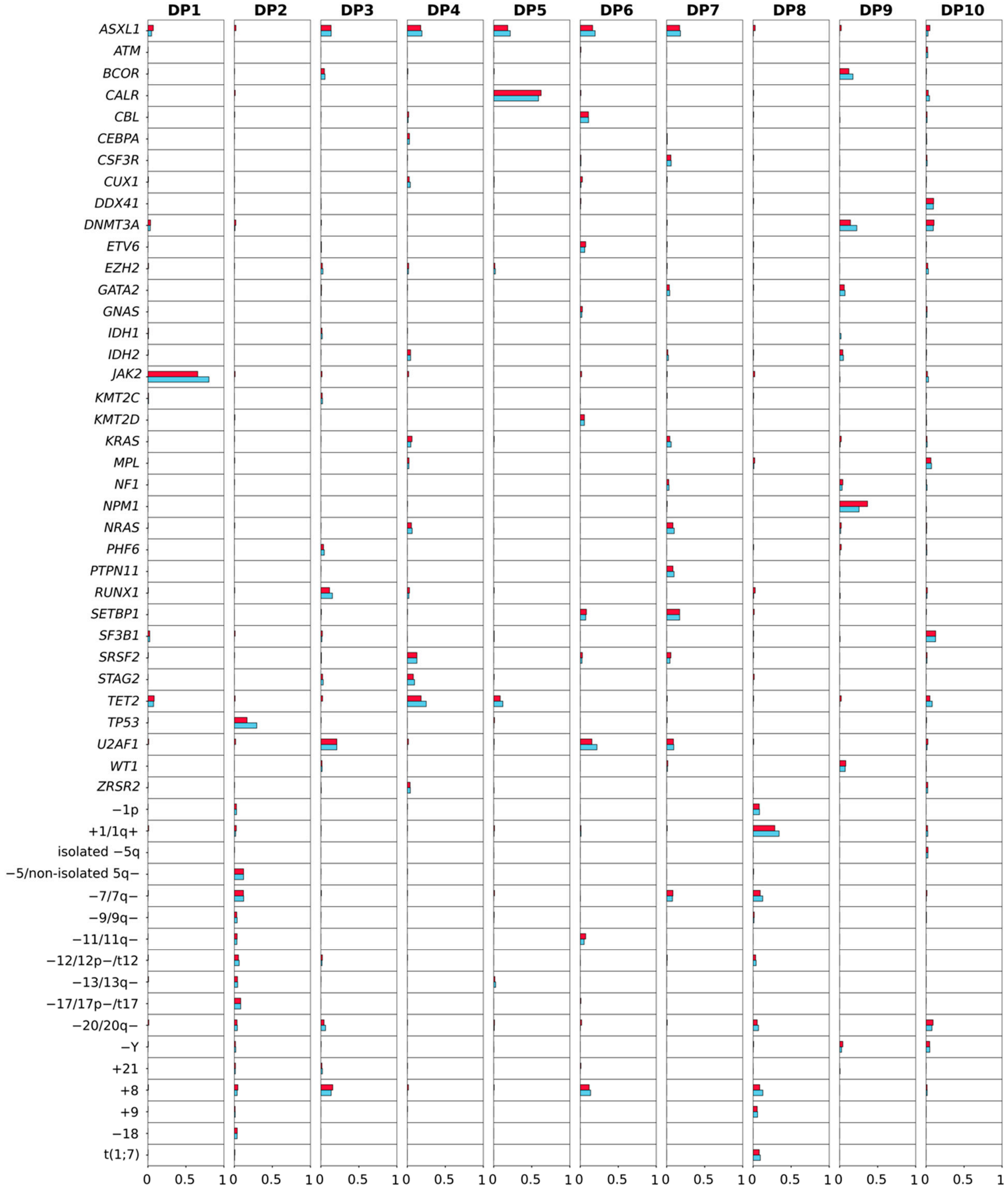

Supplementary Figure S6. Frequencies of disease phenotypes in each genomic subgroup

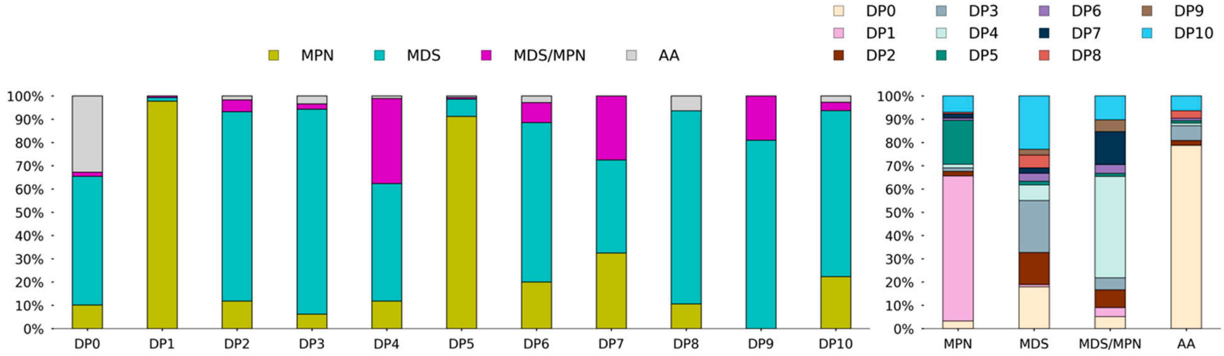

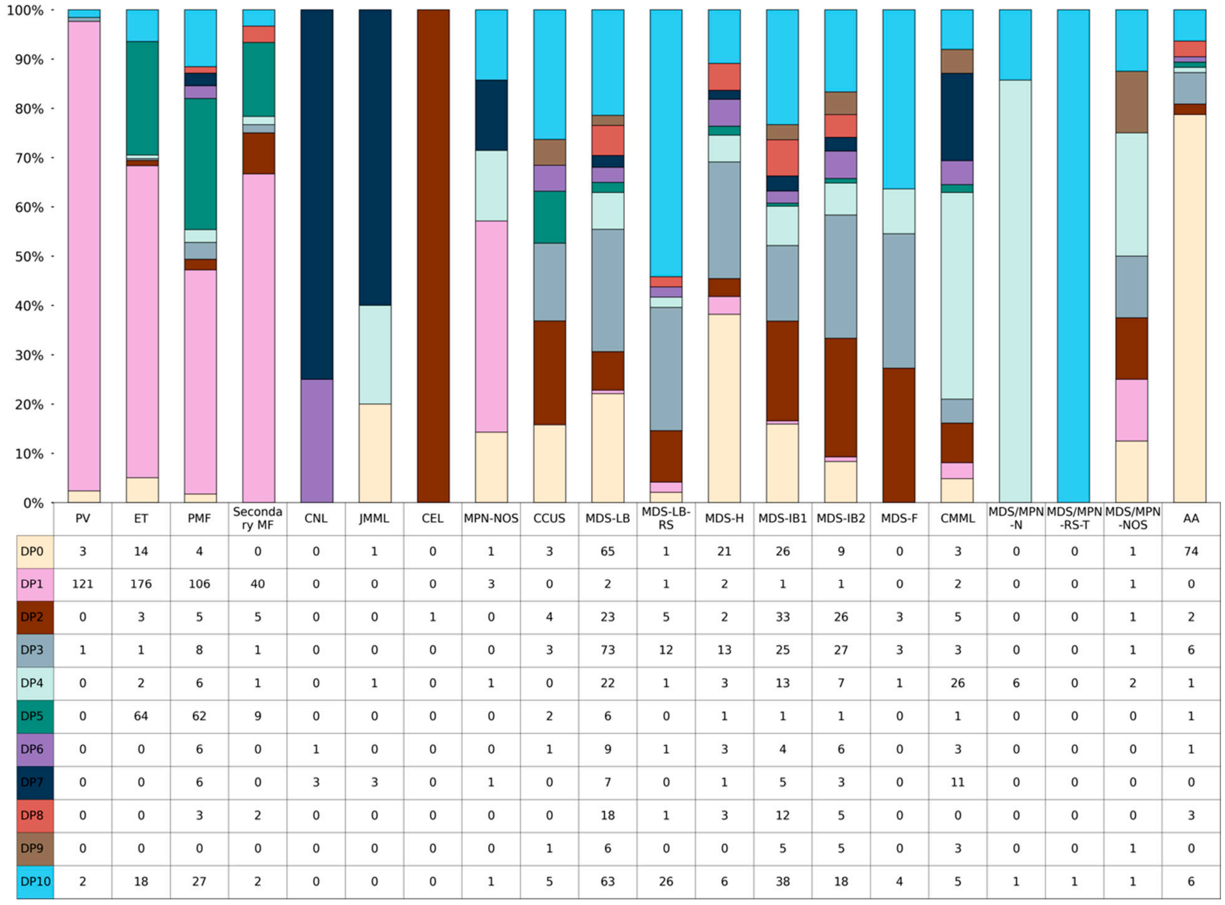

	PV	ET	PMF	Secondary MF	CNL	JMML	CEL	MPN-NOS	CCUS	MDS-LB	MDS-LB-RS	MDS-H	MDS-IB1	MDS-IB2	MDS-F	CMML	MDS/MPN-N	MDS/MPN-RS-T	MDS/MPN-NOS	AA
DP0	3	14	4	0	0	1	0	1	3	65	1	21	26	9	0	3	0	0	1	74
DP1	121	176	106	40	0	0	0	3	0	2	1	2	1	1	0	2	0	0	1	0
DP2	0	3	5	5	0	0	1	0	4	23	5	2	33	26	3	5	0	0	1	2
DP3	1	1	8	1	0	0	0	0	3	73	12	13	25	27	3	3	0	0	1	6
DP4	0	2	6	1	0	1	0	1	0	22	1	3	13	7	1	26	6	0	2	1
DP5	0	64	62	9	0	0	0	0	2	6	0	1	1	1	0	1	0	0	0	1
DP6	0	0	6	0	1	0	0	0	1	9	1	3	4	6	0	3	0	0	0	1
DP7	0	0	6	0	3	3	0	1	0	7	0	1	5	3	0	11	0	0	0	0
DP8	0	0	3	2	0	0	0	0	0	18	1	3	12	5	0	0	0	0	0	3
DP9	0	0	0	0	0	0	0	0	1	6	0	0	5	5	0	3	0	0	1	0
DP10	2	18	27	2	0	0	0	1	5	63	26	6	38	18	4	5	1	1	1	6

Supplementary Figure S7. Bradley–Terry model analysis of genetic mutation sequences in each genomic cluster

Each point corresponds to the log-odds value, indicating the relative probability that one specific genetic mutation occurs before another within a given pair. These results suggest that if the log-odds for gene B relative to gene A is 1.71, the probability of mutation B occurring first is 1.71 times greater than that of mutation A. The error bars depicted alongside each point delineate the 95% confidence intervals. We have only presented the figures of Dirichlet process (DP) groups with enough mutations for the calculation.

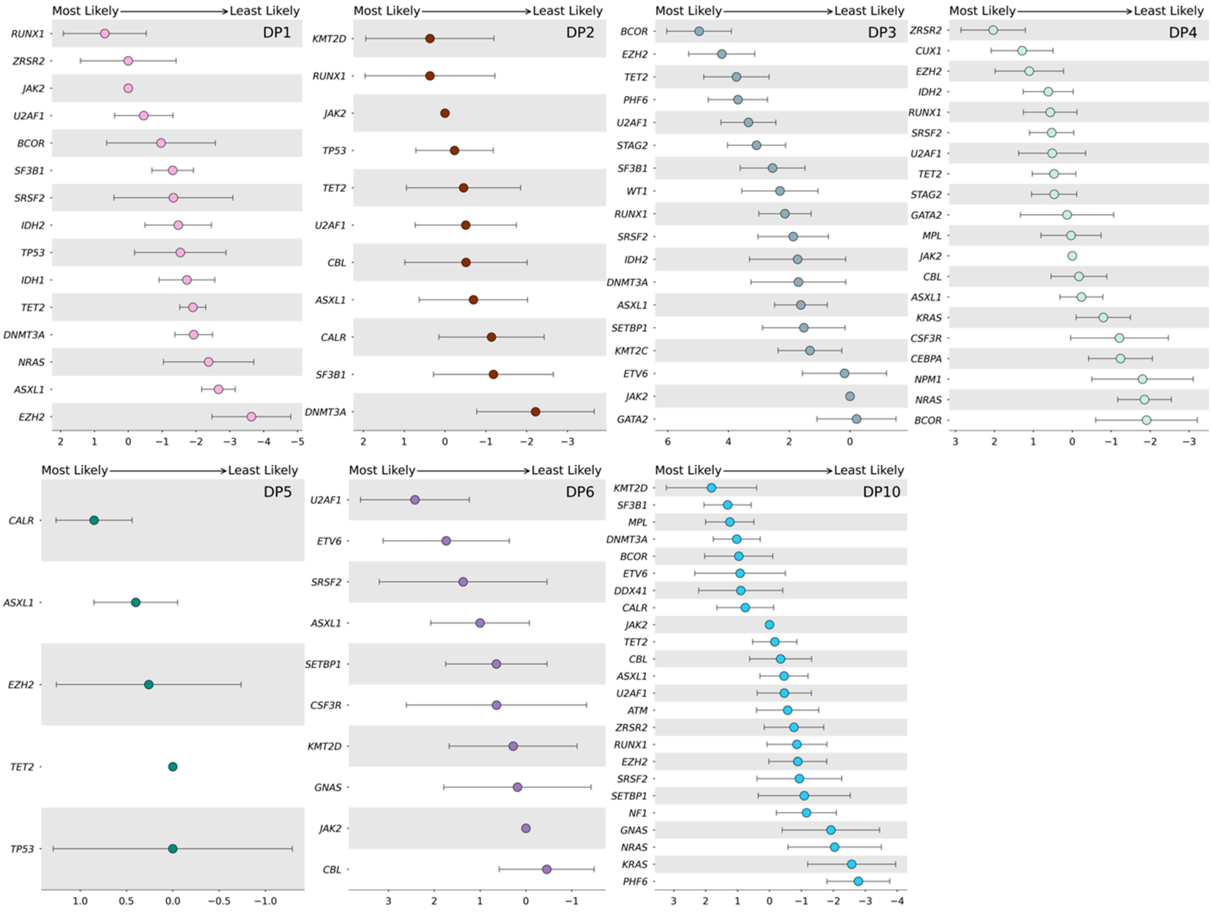

Supplementary Figure S8. Hematological parameters of each genomic subgroup

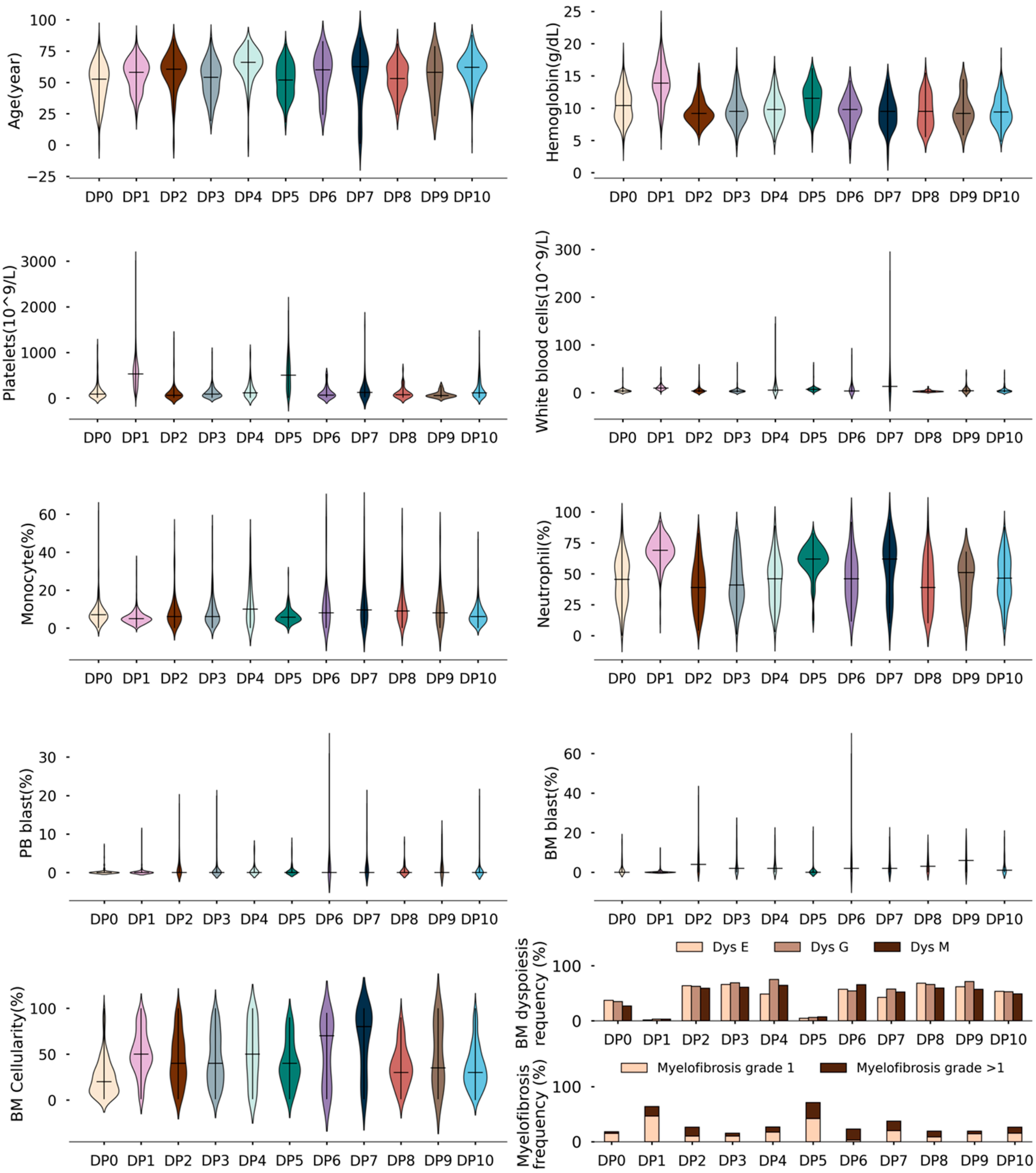

Supplementary Figure S9. Survival probability according to the newly defined genomic subgroups (training set)

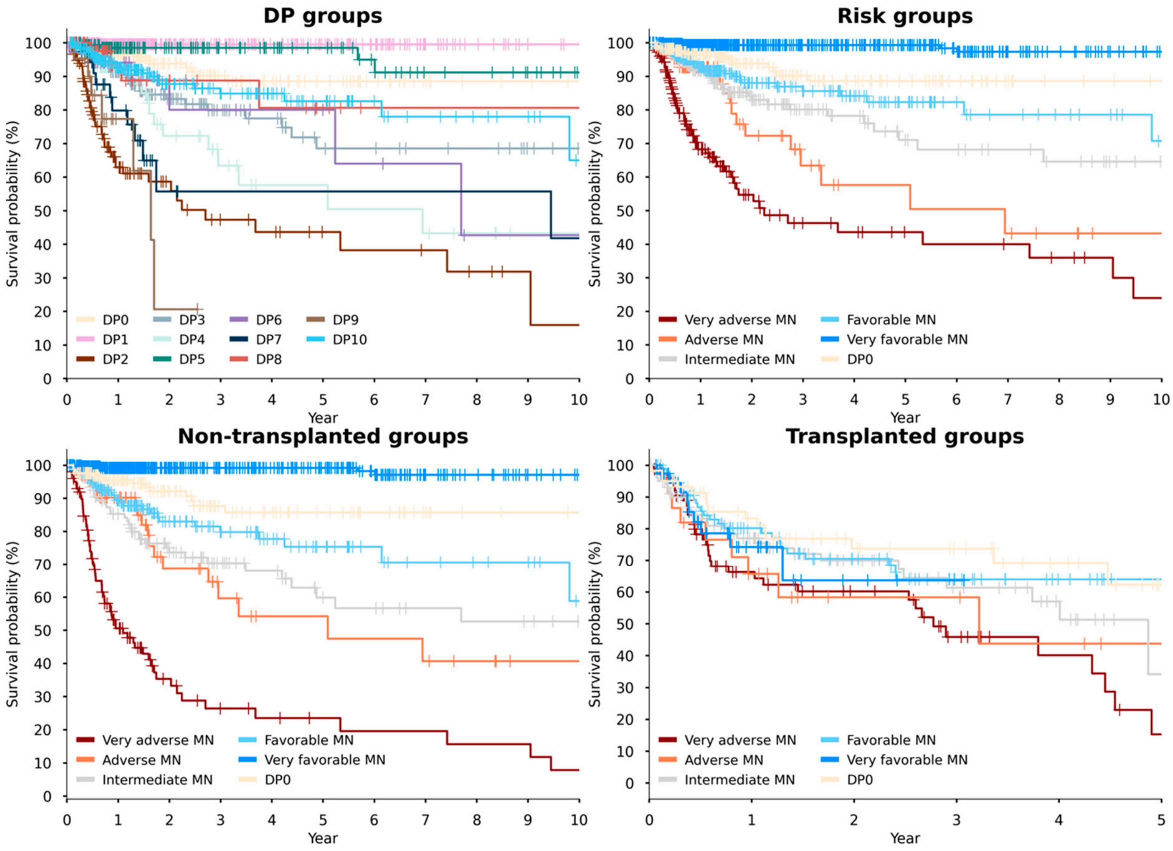

Hazard ratio 95% CI	DP0	DP1	DP2	DP3	DP4	DP5	DP6	DP7	DP8	DP9	DP10
respect to dp0	-	0.10<br>0.06 - 0.16	9.33<br>4.25 - 20.49	2.75<br>1.50 - 5.05	3.92<br>1.76 - 8.74	0.37<br>0.20 - 0.68	2.88<br>0.95 - 8.71	5.64<br>1.88 - 16.88	1.44<br>0.54 - 3.85	9.69<br>1.60 - 58.55	1.85<br>1.04 - 3.28
respect to dp1	10.20<br>6.31 - 16.51	-	95.18<br>45.86 - 197.56	28.05<br>16.47 - 47.76	39.99<br>18.97 - 84.32	3.79<br>2.24 - 6.43	29.39<br>10.11 - 85.44	57.52<br>19.99 - 165.47	14.74<br>5.78 - 37.56	98.89<br>16.77 - 583.27	18.86<br>11.51 - 30.90
respect to dp2	0.11<br>0.05 - 0.24	0.01<br>0.01 - 0.02	-	0.29<br>0.13 - 0.67	0.42<br>0.16 - 1.11	0.04<br>0.02 - 0.09	0.31<br>0.09 - 1.06	0.60<br>0.18 - 2.06	0.15<br>0.05 - 0.48	1.04<br>0.16 - 6.81	0.20<br>0.090 - 0.44
respect to dp3	0.36<br>0.20 - 0.67	0.04<br>0.02 - 0.06	3.39<br>1.50 - 7.70	-	1.43<br>0.62 - 3.28	0.14<br>0.07 - 0.26	1.05<br>0.34 - 3.24	2.05<br>0.67 - 6.28	0.53<br>0.19 - 1.44	3.53<br>0.58 - 21.61	0.67<br>0.36 - 1.25
respect to dp4	0.26<br>0.11 - 0.57	0.03<br>0.01 - 0.05	2.38<br>0.90 - 6.29	0.70<br>0.30 - 1.61	-	0.09<br>0.04 - 0.22	0.73<br>0.21 - 2.55	1.44<br>0.42 - 4.95	0.37<br>0.12 - 1.15	2.47<br>0.37 - 16.32	0.47<br>0.21 - 1.06
respect to dp5	2.69<br>1.47 - 4.92	0.26<br>0.16 - 0.45	25.10<br>11.09 - 56.81	7.40<br>3.88 - 14.11	10.55<br>4.59 - 24.21	-	7.75<br>2.51 - 23.95	15.17<br>4.96 - 46.41	3.89<br>1.42 - 10.61	26.08<br>4.26 - 159.67	4.97<br>2.69 - 9.20
respect to dp6	0.35<br>0.11 - 1.05	0.03<br>0.01 - 0.10	3.24<br>0.94 - 11.15	0.95<br>0.31 - 2.96	1.36<br>0.39 - 4.78	0.13<br>0.04 - 0.40	-	1.96<br>0.46 - 8.37	0.50<br>0.13 - 1.97	3.37<br>0.44 - 25.77	0.64<br>0.21 - 1.95
respect to dp7	0.18<br>0.06 - 0.53	0.02<br>0.01 - 0.05	1.65<br>0.49 - 5.64	0.49<br>0.16 - 1.49	0.70<br>0.20 - 2.39	0.07<br>0.02 - 0.20	0.51<br>0.12 - 2.18	-	0.26<br>0.07 - 0.99	1.72<br>0.23 - 13.09	0.33<br>0.11 - 0.99
respect to dp8	0.69<br>0.26 - 1.85	0.07<br>0.03 - 0.17	6.46<br>2.10 - 19.87	1.90<br>0.70 - 5.21	2.71<br>0.87 - 8.44	0.26<br>0.09 - 0.70	1.99<br>0.51 - 7.82	3.90<br>1.00 - 15.19	-	6.71<br>0.94 - 48.09	1.28<br>0.48 - 3.43
respect to dp9	0.10<br>0.02 - 0.62	0.01<br>0.00 - 0.06	0.96<br>0.15 - 6.31	0.28<br>0.05 - 1.74	0.40<br>0.06 - 2.67	0.04<br>0.01 - 0.23	0.30<br>0.04 - 2.28	0.58<br>0.08 - 4.43	0.15<br>0.02 - 1.07	-	0.19<br>0.03 - 1.16
respect to dp10	0.54<br>0.30 - 0.96	0.05<br>0.03 - 0.09	5.05<br>2.28 - 11.17	1.49<br>0.80 - 2.76	2.12<br>0.94 - 4.76	0.20<br>0.11 - 0.37	1.56<br>0.51 - 4.74	3.05<br>1.01 - 9.18	0.78<br>0.29 - 2.10	5.24<br>0.86 - 31.79	-

Supplementary Figure S10. Survival probability according to the newly defined genomic subgroups (validation set)

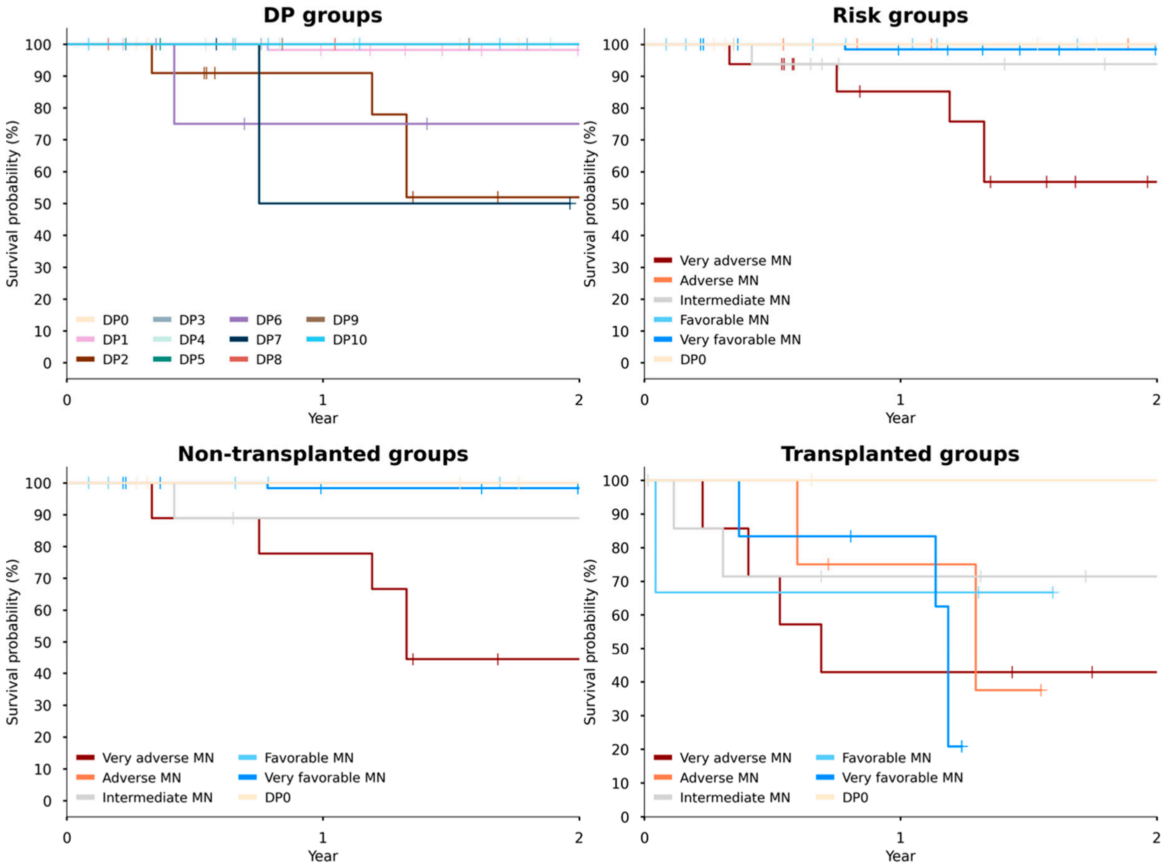

Hazard ratio 95% CI	Adverse	Favorable	Inermediate	Very adverse	Very favorable
respect to adverse	-	0.15<br>0.01 - 2.14	0.52<br>0.04 - 7.48	2.24<br>0.13 - 37.78	0.14<br>0.013 - 1.56
respect to favorable	6.53<br>0.47 - 91.28	-	3.37<br>0.52 - 21.64	14.64<br>1.85 - 115.99	0.94<br>0.23 - 3.87
respect to intermediate	1.94<br>0.13 - 28.14	0.30<br>0.05 - 1.91	-	4.35<br>0.52 - 36.12	0.28<br>0.06 - 1.23
respect to very adverse	0.45<br>0.03 - 7.52	0.07<br>0.01 - 0.54	0.23<br>0.03 - 1.91	-	0.06<br>0.01 - 0.37
respect to very favorable	6.97<br>0.64 - 75.99	1.07<br>0.26 - 4.41	3.59<br>0.81 - 15.91	15.63<br>2.74 - 89.21	-

Supplementary Figure S11. Comparison of the survival probabilities between hematopoietic stem cell transplantation (HSCT) and non-HSCT patients according to the risk categories

The number of asterisks denotes the P value (*P<0.05, and ***P<0.01) for comparisons between HSCT and non-HSCT patients, in each group

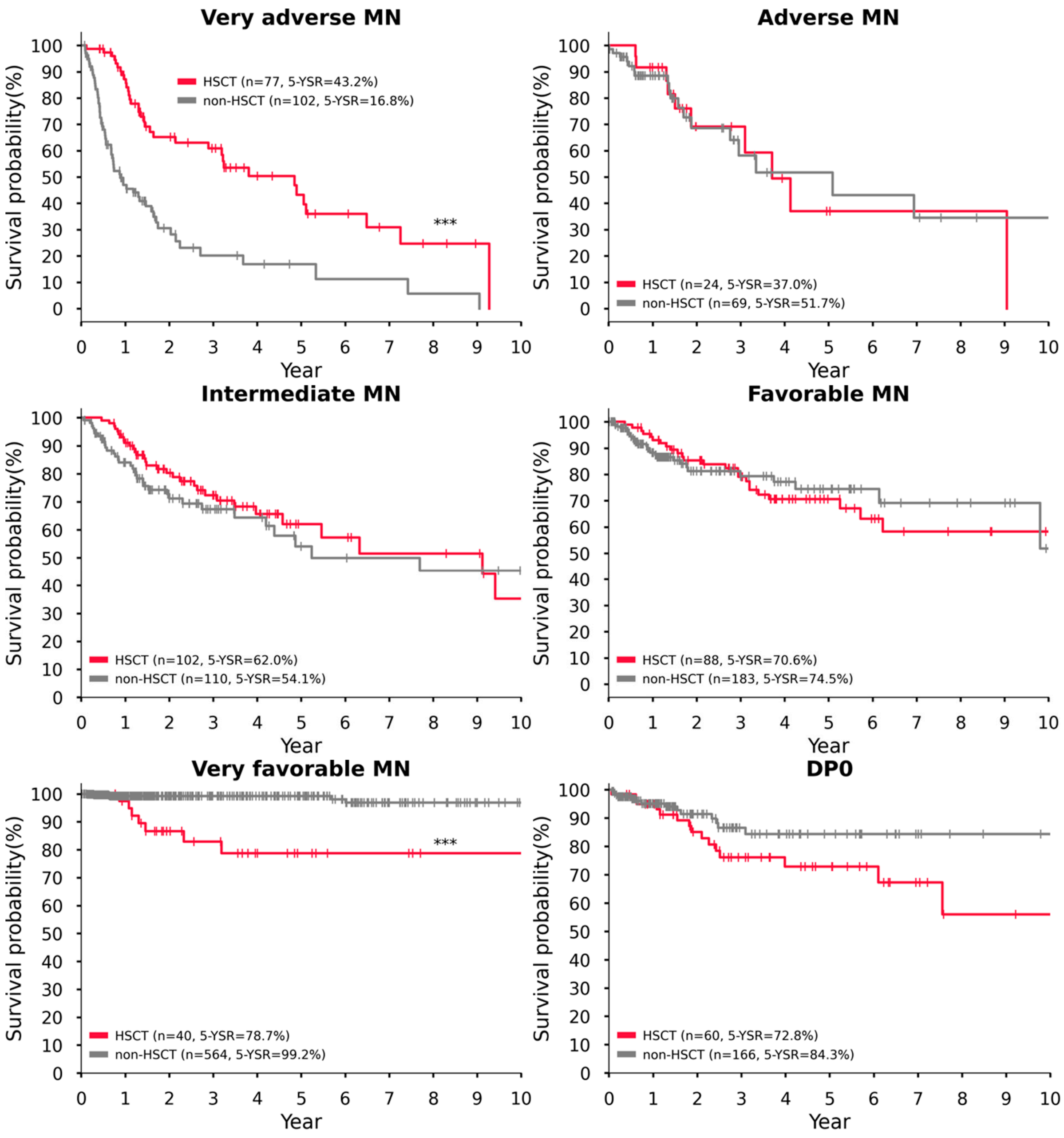

Supplementary Figure S12. Comparison of the survival probabilities between hematopoietic stem cell transplantation (HSCT) and non-HSCT patients according to the genomic subgroups

The number of asterisks denotes the P value (*P<0.05, and ***P<0.01) for comparisons between hematopoietic stem cell transplantation (HSCT) and non-HSCT patients, in each group

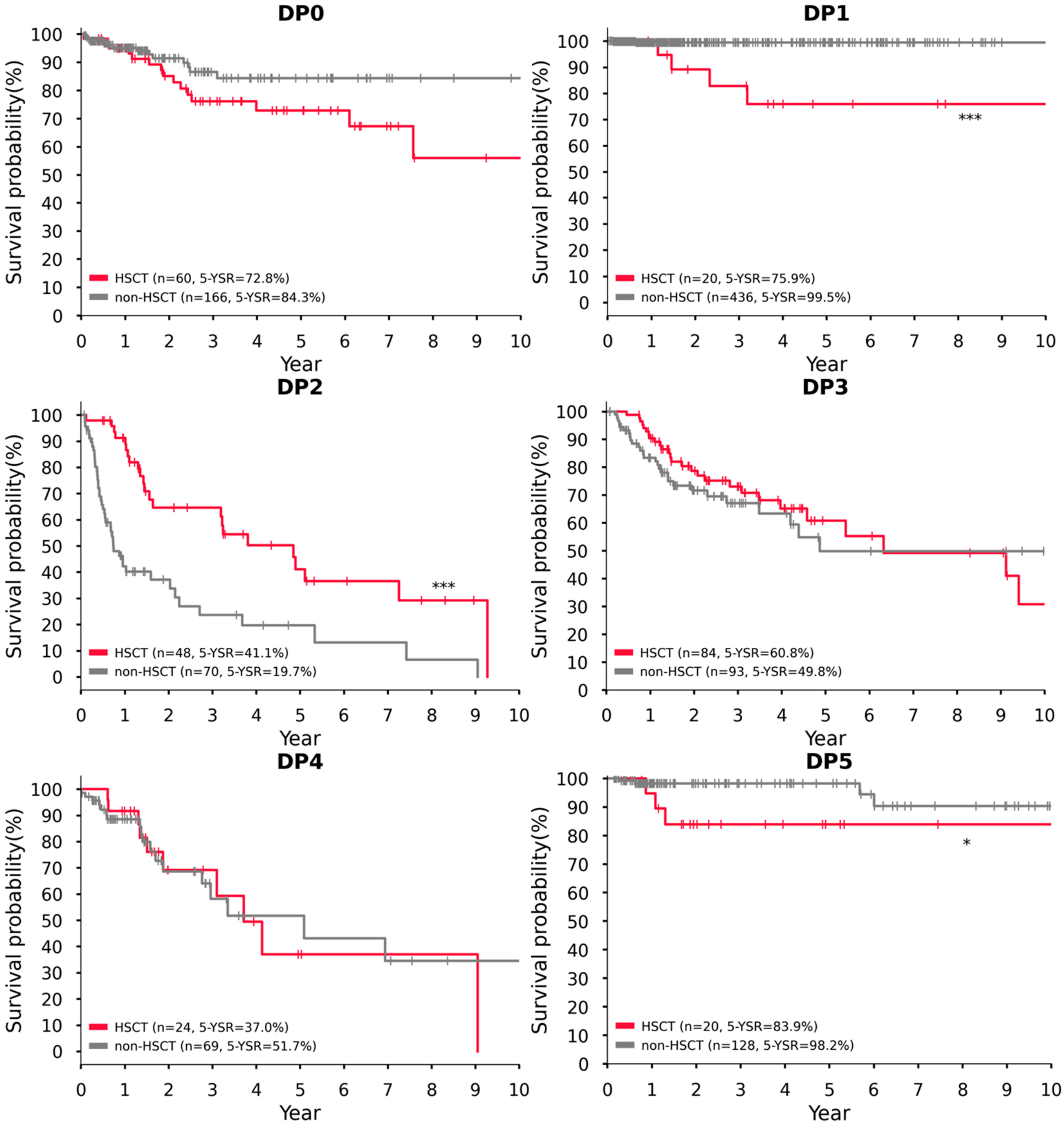

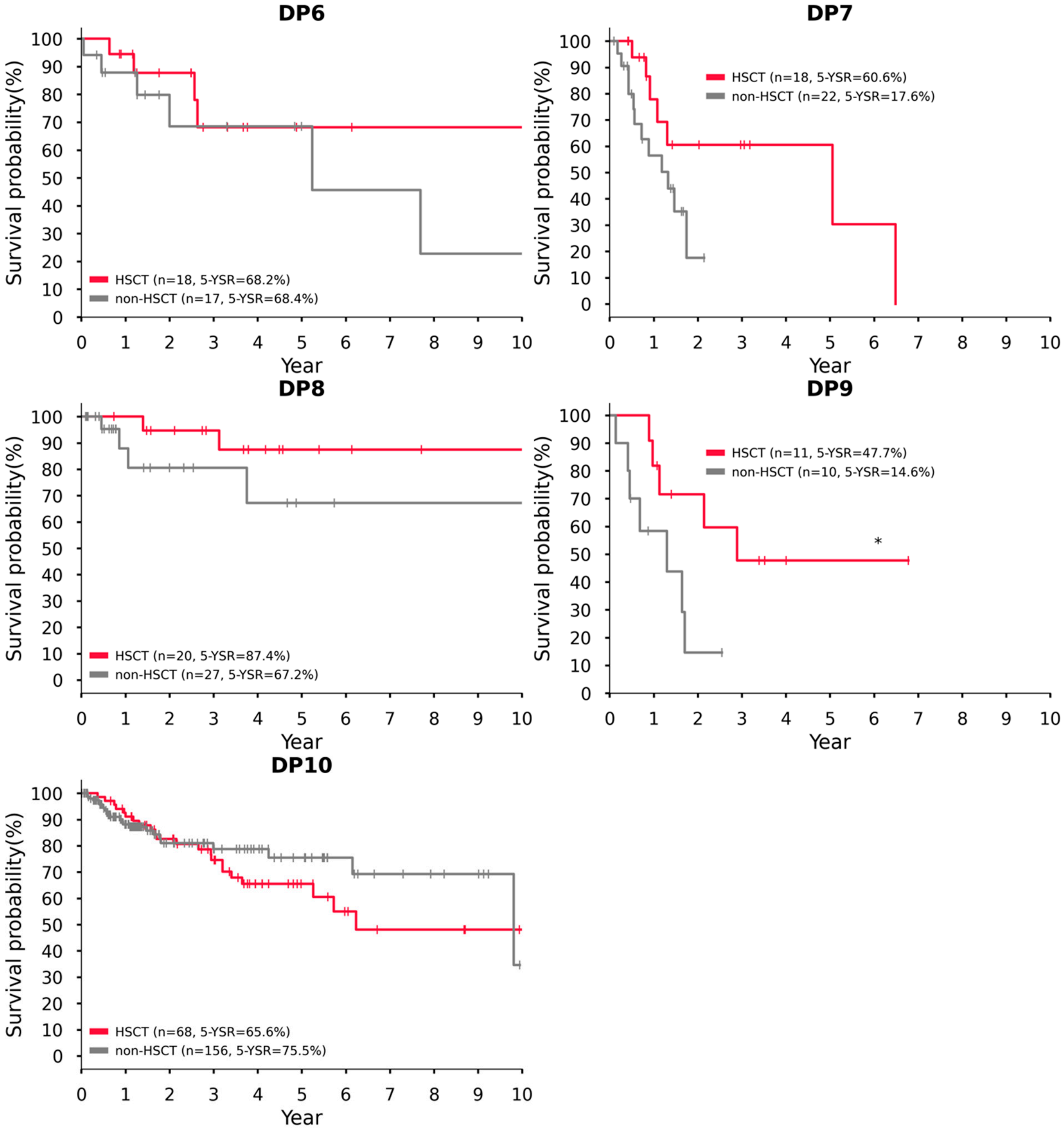
DP6
Survival probability(%)
Year
HSCT (n=18, 5-YSR=68.2%)
non-HSCT (n=17, 5-YSR=68.4%)
DP7
Survival probability(%)
Year
HSCT (n=18, 5-YSR=60.6%)
non-HSCT (n=22, 5-YSR=17.6%)
DP8
Survival probability(%)
Year
HSCT (n=20, 5-YSR=87.4%)
non-HSCT (n=27, 5-YSR=67.2%)
DP9
Survival probability(%)
Year
HSCT (n=11, 5-YSR=47.7%)
non-HSCT (n=10, 5-YSR=14.6%)
*
DP10
Survival probability(%)
Year
HSCT (n=68, 5-YSR=65.6%)
non-HSCT (n=156, 5-YSR=75.5%)

Supplementary Figure S13. Validation of genomic risk categories compared with IPSS-R and IPSS-M in MDS

Among the 405 patients diagnosed with MDS and available scores from IPSS-R and IPSS-M, our system demonstrated a lower concordance index (C-index 0.666) compared to IPSS-R (0.778) and IPSS-M (0.818). Even after reanalyzing the data for patients who did not undergo transplantation (n=208), IPSS-M maintained the highest predictive accuracy (0.828), followed by IPSS-R (0.770) and our risk category (0.669)

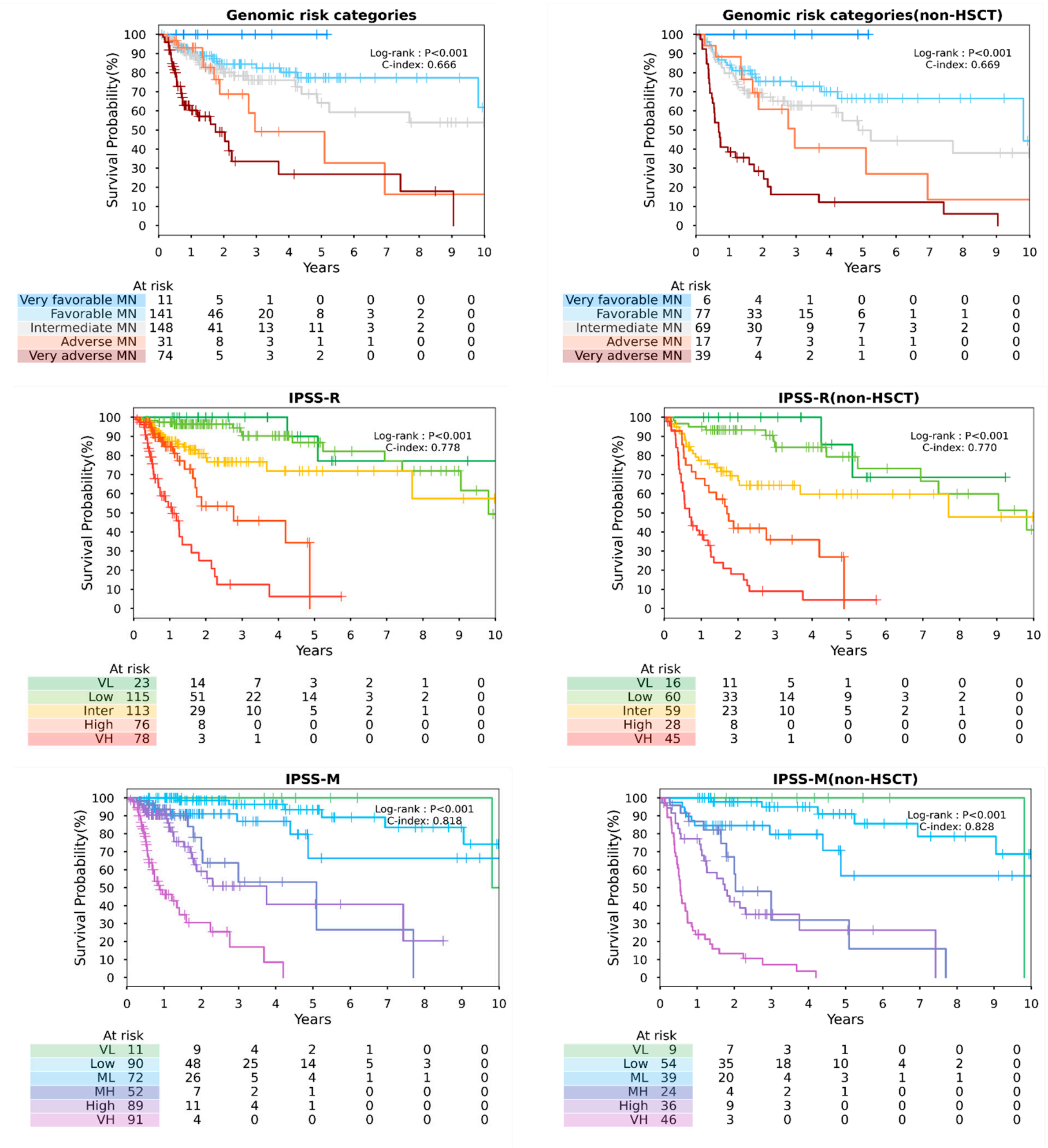

References

1. Lee, J.M.; Kim, Y.J.; Park, S.S.; Han, E.; Kim, M.; Kim, Y. Simultaneous Monitoring of Mutation and Chimerism Using Next-Generation Sequencing in Myelodysplastic Syndrome. *J Clin Med* **2019**, *8*, doi:10.3390/jcm8122077.
2. Lee, J.M.; Lee, H.; Eom, K.S.; Lee, S.E.; Kim, M.; Kim, Y. Impact of Integrated Genetic Information on Diagnosis and Prognostication for Myeloproliferative Neoplasms in the Next-Generation Sequencing Era. *J Clin Med* **2021**, *10*, doi:10.3390/jcm10051033.
3. Li, M.M.; Datto, M.; Duncavage, E.J.; Kulkarni, S.; Lindeman, N.I.; Roy, S.; Tsimberidou, A.M.; Vnencak-Jones, C.L.; Wolff, D.J.; Younes, A.; et al. Standards and Guidelines for the Interpretation and Reporting of Sequence Variants in Cancer: A Joint Consensus Recommendation of the Association for Molecular Pathology, American Society of Clinical Oncology, and College of American Pathologists. *J Mol Diagn* **2017**, *19*, 4-23, doi:10.1016/j.jmoldx.2016.10.002.
4. Horak, P.; Griffith, M.; Danos, A.M.; Pitel, B.A.; Madhavan, S.; Liu, X.; Chow, C.; Williams, H.; Carmody, L.; Barrow-Laing, L.; et al. Standards for the classification of pathogenicity of somatic variants in cancer (oncogenicity): Joint recommendations of Clinical Genome Resource (ClinGen), Cancer Genomics Consortium (CGC), and Variant Interpretation for Cancer Consortium (VICC). *Genet Med* **2022**, *24*, 986-998, doi:10.1016/j.gim.2022.01.001.
